# A prospective investigation of whether parent psychopathology explains the relationship between parent maltreatment and offspring mental health

**DOI:** 10.1017/S0954579425101120

**Published:** 2026-01-23

**Authors:** Joanna Young, Catherine Harris, Kellie Courtney, Cathy Spatz Widom

**Affiliations:** 1 PTSD Program, Stratton VA Medical Center, Albany, NY, USA; 2 Graduate Center, City University of New York, NewYork, NY, USA; 3 Psychology Department, John Jay College, City University of New York, NewYork, NY, USA

## Abstract

Previous research reports that offspring of parents with histories of childhood maltreatment are at increased risk for mental health problems, yet the mechanisms remain unclear. This study examines the extent to which parent psychopathology mediates the relationship between parent maltreatment history and offspring psychopathology. Using a prospective cohort design, individuals with documented histories of childhood maltreatment (ages 0 – 11 years) during 1967 – 1971 and demographically matched controls were followed into adulthood and first interviewed in 1989-1995 (*N* = 1,196). Offspring (*N* = 697, Mage = 22.8 years) were assessed in 2009 – 2010. A general p-factor structure and a model with specific latent constructs were tested. Structural equation modeling was used for mediation. The results indicated that only minor offspring of maltreated parents exhibited more symptoms of depression than offspring of controls. Parent psychopathology predicted offspring psychopathology. Parent depression and dysthymia predicted greater offspring depression, anxiety, PTSD, alcohol use, drug use, and marijuana use. Parent anxiety and alcohol and drug symptoms also predicted offspring alcohol symptoms. There was no evidence that parent psychopathology explained the relationship between a parent’s history of maltreatment and their offspring’s psychopathology. These new results suggest that reconsideration of some assumptions about the intergeneration impacts of maltreatment may be warranted.

## Introduction

The long-term consequences of childhood maltreatment extend beyond the individual child, often affecting the next generation. Increasing evidence suggests that child and adolescent offspring of parents with histories of childhood maltreatment are at increased risk for mental health problems (Bosquet Enlow et al., 2018; Collishaw et al., 2007; Delker et al., 2014, Miranda et al., 2011; 2013a). Emotional and behavioral problems experienced by these offspring in adolescence frequently persist and may extend into adulthood (Roberts et al., 2015). Understanding the factors that may explain the relationship between a parent’s history of childhood maltreatment and their offspring’s mental health is important for developing effective strategies to treat and prevent the intergenerational effects of childhood maltreatment.

One potential mechanism that may explain the relationship between a parent’s maltreatment history and their offspring mental health is through the parents’ own psychopathology. Childhood maltreatment has been associated with many types of mental health problems in adulthood, including depression, anxiety, posttraumatic stress disorder (PTSD), substance abuse, and personality disorders (Baldwin et al., 2023; Battle et al., 2004; Chandan et al., 2019; Collishaw et al., 2007; Gilbert et al., 2009; Grummitt et al., 2024; Guiney et al., 2024; Kisely et al., 2018; Scott et al., 2012). If parent history of childhood maltreatment increases risk for parent psychopathology, the parent psychopathology may impact the well-being of their offspring, including offspring psychopathology. For example, parents with depression may experience mood changes, model ineffective behaviors, or inadvertently increase their offspring’s exposure to stress. Parents struggling to cope with the psychological impact of their own abuse history may have difficulty supporting the emotional development of their children, which may in turn leave their children vulnerable to developing psychological symptoms and/or disorders.

Prior research exploring the extent to which parent psychopathology explains the relationship between a parent’s maltreatment history and offspring mental health has yielded mixed findings. Some studies have found that parent psychopathology mediates the relationship between parent childhood maltreatment history and offspring mental health (Koverola et al., 2005; Madigan et al., 2015, Miranda et al., 2013a; 2013b; Morrel et al., 2003; Plant et al., 2017; Roberts et al., 2015), though these focus heavily on maternal depression. Other studies have *not* found that maternal mental health serves as a mediator (Miranda et al., 2011; Thompson, 2007).

A number of possible reasons may explain the inconsistencies in the findings thus far and make comparisons across existing studies challenging. Specifically, studies vary in terms of (1) assessments of parent psychopathology, whether using global or specific symptom clusters; (2) criteria for determining child maltreatment in parents; (3) the sex of the parent studied; (4) assessment of offspring psychopathology, including methods, sources, and age when assessed; (5) ways of dealing with comorbidity in the forms of psychopathology; and (6) study designs (longitudinal versus cross-sectional). We briefly review these variations and discuss how they are relevant to existing findings.

### Assessing parent psychopathology as a mediator

One possible reason for the inconsistent findings in prior research is that studies have used varying degrees of specificity in assessing parents’ psychopathology. Some studies have focused on specific psychiatric disorders, whereas other examine broad indicators of psychological distress. For example, studies have found support for the potential mediating role of depression in the relationship between parent maltreatment history and offspring mental health (Choi et al., 2019; Gong et al., 2024, Miranda et al., 2013a; 2013b; Plant et al., 2017; Roberts et al., 2015). Miranda et al. (2013a; 2013b) found that maternal depressive symptoms significantly mediated the relationship between maternal maltreatment history and offspring outcomes. This is consistent with evidence that maternal depression may disrupt healthy parent – child interactions (Humphreys et al., 2018) and that depression may lead parents to engage in parenting styles that are hostile, detached, or inconsistent (Goodman, 2020; Sellers et al., 2014). Although many of these studies exclusively examined parent *depressive* symptoms, other studies investigated the potential mediating role of parent generalized anxiety (Shih et al., 2023) and alcohol and/or drug abuse or dependence. Together, these studies suggest that examining specific forms of parent psychopathology may be beneficial. It is possible that using global indicators of parents’ psychological distress or general mental health may mask significant mediational effects of specific symptoms or specific disorders.

Support for examining specific disorders rather than focusing exclusively on transdiagnostic mechanisms comes from theories that posit disorder-specific processes as mechanisms linking psychopathology and impairment over the lifespan and posit that these processes may play a role in pathways from parent to offspring psychopathology. For example, stress generation theory (Hammen, 1991) initially posited a disorder-specific process through which individuals with depression are more likely to experience stressful life events which increase the person’s risk of experiencing future depression. A recent meta-analysis of prospective longitudinal studies testing the stress generation theory also found evidence of disorder-specific effects, with unique patterns of effects found for depression relative to anxiety. Other studies have found that parent PTSD specifically predicts offspring PTSD when controlling for other anxiety disorders and depression (Starr et al., 2014; Yehuda et al., 2001). Thus, examining specific parent disorders appears to be supported by both recent theories and recent empirical work.

Studies have also been limited by their reliance on categorical diagnoses, rather than examining continuous symptoms or dimensions of severity. These studies exclude potentially important subthreshold symptoms that may be associated with severe functional impairment, but which do not rise to the level of meeting criteria for a diagnosis. Multiple studies have highlighted the need for future work that incorporates dimensional variables as mediators (Ethridge et al., 2022; Starr et al., 2014).

In contrast to disorder-specific approaches, transdiagnostic models, such as the general *p*-factor (Caspi et al., 2014) and Hi-TOP (Kotov et al., 2017), conceptualize mental disorders as overlapping continuous dimensions, with higher-order factors representing patterns of comorbidity between multiple disorders (Clark et al., 2024). While transdiagnostic models have become increasingly popular in recent years, the use of such broad conceptualizations of psychopathology has been controversial. Although some argue that the *p*-factor represents the effects of shared etiological factors (Lahey et al., 2011; Stochl et al., 2015), others suggest that it represents a nonspecific impairment that follows from problems that do not necessarily share anything with each other, etiologically or conceptually (Widiger & Oltmanns, 2017; van Bork et al., 2017). Both approaches may prove to be informative in terms of determining mechanisms that may explain the role of parental psychopathology on offspring mental health.

### Criteria for determining child maltreatment of parents

Prior studies have used different methods to define or operationalize key variables. For example, studies have used different approaches to ascertaining whether the parent has a history of childhood maltreatment, with most prior studies relying on parents’ retrospective self-reports (Roberts et al., 2015). Prospective data can be obtained through court documents, child protective services, or hospital records (Baldwin et al., 2019). Retrospective data are based on self-report questionnaires and interviews and may be ascertained at any age including adulthood. Both methods have shortcomings; retrospective studies are subject to memory biases and increased association with psychopathology (Danese & Widom, 2024), whereas prospective studies are missing unreported cases.

It is important to consider whether these studies are using retrospective or prospective data to operationalize childhood maltreatment, as prior studies have found differences specifically in how they relate to psychopathology (Danese & Widom, 2024) and that offspring of female parents with substantiated cases of childhood maltreatment are more likely to be maltreated compared to offspring of parents without substantiated cases (Bartlett et al., 2017). The current literature lacks studies with substantiated cases of parent childhood maltreatment history and primarily relies on retrospective reports of childhood maltreatment, which has found support for parent psychopathology as a mediator in the relationship between parent maltreatment history and their offspring’s mental health outcomes (2013b; Choi et al., 2019; Gong et al., 2024, Miranda et al., 2013a; Plant et al., 2017; Roberts et al., 2015; Shih et al., 2023).

### Differences as a function of parent sex

The impact of parent maltreatment history and parent psychopathology on offspring mental health may differ depending on the parent’s sex. The existing literature has been overwhelmingly focused on maternal abuse history and maternal psychopathology. To date, very few studies have focused on fathers, although there is increasing interest in the paternal role. Research suggests that paternal abuse history may have lasting effects on parenting behavior (Ehrensaft et al., 2015; Zanoni et al., 2014), and there is evidence that paternal psychopathology may impact offspring psychopathology and have a unique or additive role (Carro et al., 1993; Connell & Goodman, 2002; Fletcher et al., 2011; Giallo et al., 2014; Ramchandani et al., 2005, 2008). However, there are a lack of studies that examine how paternal psychopathology may act as a potential mediator in the relationship between parent childhood maltreatment history and offspring mental health, as many studies focus specifically on the role of maternal depression.

### Assessment of offspring psychopathology as an outcome

In prior research, there has also been wide variability in the level of specificity at which offspring psychopathology symptoms were assessed. Many studies have conceptualized offspring mental health in terms of broader dimensions, such as internalizing and externalizing behaviors (Choi et al., 2019; Collishaw et al., 2007; Koverola et al., 2005; Russotti et al., 2021), whereas others have focused on more specific offspring disorders (Plant et al., 2013; Roberts et al., 2015). Some studies have found evidence of mediation through specific parent disorders to offspring internalizing symptoms but not externalizing symptoms (Koverola et al., 2005; Russotti et al., 2021), suggesting that maternal psychopathology may not serve as a universal mediator across different domains of offspring psychopathology. Still others have focused on even broader dimensions of offspring mental health, without distinguishing between internalizing and externalizing disorders/symptoms (Bosquet Enlow et al., 2018).

Whether a general or specific approach is taken to measuring offspring psychopathology may be important to consider because there is evidence of greater symptom differentiation across development, with broader dimensions typically favored in early childhood and more specific disorders developing later in life, particularly as adolescents approach adulthood (Caspi et al., 2014; Murray et al., 2016; Patalay et al., 2015). This phenomenon, which has been referred to as *p*-*differentiation*, has implications for how psychopathology variables are assessed in samples of different ages. Inconsistent findings in prior studies may be due to the lack of fit between the level of specificity at which the psychopathology variables were tested and the ages of those who comprised the study sample(s).

### Different sources used to assess offspring psychopathology

Studies have also utilized different sources of information when assessing offspring outcomes (e.g., parent report, teacher report, etc.). A majority of studies have relied on parents’ reports to assess offspring psychopathology, whereas relatively few studies have assessed offspring psychopathology using offspring’s self-reports. This is problematic because parent ratings of their offspring may be biased by the parents’ own psychopathology at the time of the assessment (Maoz et al., 2014; Najman et al., 2000; Zhang & Ji, 2024). In some studies that have used the reports of both parent and offspring to assess offspring outcomes, mediation is found for parent psychopathology, but *only* when using parents’ reports, and not when using offspring self-reports.

Other studies suggest that parents’ reports may be biased when assessing offspring externalizing symptoms or behaviors. For example, Choi et al. (2019) and Russotti et al. (2021) both tested very similar models linking maternal maltreatment history to offspring internalizing/externalizing symptoms through maternal depression, but findings differed. Using parent and teacher reports of offspring symptoms, Choi et al. (2019) found an indirect pathway through maternal depression to both offspring internalizing and externalizing symptoms. Using offspring self-report, Russotti et al. (2021) found a pathway *only* to offspring internalizing. More research is needed in which offspring outcomes are assessed via offspring self-report.

### Differences in age(s) at which offspring were assessed

The impact of parental psychopathology on offspring mental health may also depend on the age of the offspring at the time of the assessment. Offspring may vary in how susceptible they are to risk factors related to their parent’s abuse history or their parent’s psychopathology at different parts of the life span. Age differences in offspring outcomes may be due to sensitive periods, in which specific risk factors have especially strong influences due to the developmental needs of that period (Cicchetti & Rogosch, 2002; Cicchetti, 1993). For example, the sensitive window hypothesis posits that maternal depression during the first year of the child’s life is particularly impactful on the child’s mental health, above and beyond exposure(s) at later periods in life (Bagner et al., 2010; Bureau et al., 2009). Others have found that maternal depression exerts stronger effects over the first 15 years of the child’s lifetime (Hammen et al., 2008). Although studies have varied with respect to the specific timing and length of these sensitive periods, research generally suggests that offspring are most vulnerable to being negatively impacted by their parents’ psychopathology when exposed at earlier stages in life (Goodman & Gotlib, 1999).

While offspring may be more vulnerable to the effects of their parent’s psychopathology at younger ages, they are more likely to manifest mental health symptoms during adolescence (Elsayed et al., 2019). Studies of intergenerational effects have primarily assessed offspring in early and middle childhood (Marceau et al., 2022). As a result, many studies have assessed offspring well before the age when symptoms (e.g., depression) are likely to manifest (Goodman, 2020). For example, in one study where maternal depression was *not* found to be a significant mediator, offspring were assessed in early childhood at age 4 (Thompson, 2007), rather than in adolescence or adulthood. The situation may be further compounded by the fact that different disorders are associated with different ages of onset, with much earlier ages of onset found for anxiety disorders (age 11) than for mood disorders (age 30) and substance use (age 20)(Kessler et al., 2005). Thus, the lack of significant findings in these studies may be due to complications of study design and a focus on examining offspring mental health at a time when their symptoms would not have been expected to manifest or be measurable.

Despite recent advances in theoretical and conceptual understandings of offspring psychopathology and trajectories of psychological development, researchers have tended to study offspring either in one developmental period – which may produce findings that do not generalize to other periods in the children’s lives – or in such a broad age range that multiple age groups and developmental stages are included in one category – which likely masks important developmental issues (Goodman, 2020). Overall, there are few studies assessing offspring at older ages, and therefore, a gap in knowledge about whether offspring problems persist beyond the adolescent period.

### Issues of comorbidity

Some studies assessing adolescent samples suggest that the intergenerational transmission of psychopathology occurs via comorbid symptom severity, rather than by domain-specific symptoms or disorders. However, adolescence is a period when comorbidity is especially prominent (Beauchaine & McNulty, 2013), as many disorders emerge during this period, and it is unclear whether the same intergenerational mechanisms would apply if offspring were assessed in adulthood. In prior studies, these comorbidities have been accounted for either by focusing on broader dimensions of psychopathology, within which multiple comorbid disorders are subsumed, or by controlling for potential areas of overlap (i.e., incremental validity) when assessing specific disorders.

### Cross-sectional versus longitudinal designs

Finally, in studies that have assessed parent and offspring psychopathology symptoms contemporaneously in cross-sectional studies, it is impossible to determine the direction of effects. This is the case regardless of whether parents’ or offspring’s reports were used to assess offspring symptoms.

In sum, despite inconsistencies in the literature, the studies reviewed here suggest that a parent’s history of childhood maltreatment and subsequent psychopathology may impact their offspring’s mental health and that parental abuse history may exert its effects on offspring psychopathology, in part, through the parents’ own psychopathology. The present study aims to fill some of the gaps in the existing literature and contribute to better understanding of this phenomenon.

### The current study

The current study examines the role of parents’ psychopathology as a potential mediator in explaining the relationship between parents’ maltreatment history and offspring’s psychopathology. We use data from a longitudinal study that followed a large group of children with documented cases of maltreatment and demographically matched controls and interviewed both groups over multiple time points including into middle adulthood. The current work focuses on individuals who became parents and who, along with their offspring, were assessed in 2009 – 2010 as part of a study of the intergenerational transmission of abuse and neglect (Widom et al., 2015).

Using a prospective cohort design, children with documented histories of childhood abuse and neglect prior to age 11 (during 1967 – 1971) were matched with a group of non-maltreated children and followed into adulthood when many of them became parents. Parent psychopathology variables, including depression, dysthymia, anxiety, PTSD, and alcohol/drug use, were assessed in young adulthood (parents’ mean age = 28.9) through structured diagnostic interviews administered during 1989 – 1995. To address the omission of fathers in prior studies of parent psychopathology as potential mediators of the relationship between parent maltreatment and offspring psychopathology, both mothers and fathers were included in the parent sample studied here.

### Hypotheses

We have three main hypotheses: (1) Parents with a history of childhood maltreatment will report more symptoms of psychiatric disorders than parents without maltreatment histories; (2) Offspring of parents with a history of childhood maltreatment will report more psychological symptoms than offspring of matched controls; and (3) Parent psychopathology (depression, dysthymia, anxiety, PTSD, and alcohol and drug abuse and/or dependence symptoms) will partially mediate the relationship between parental maltreatment history and offspring psychopathology. Because of potential differences in consequences for offspring at different stages of development and because of the need to use developmentally appropriate measures, we consider the role of parent maltreatment on minor and adult offspring separately. Given the dearth of prior literature on male parents, we make no specific predictions about whether (or how) paternal childhood maltreatment and paternal psychopathology will influence offspring mental health, compared to the influence of mothers on offspring mental health.

We have adopted multiple modeling approaches to examining these relationships. Psychiatric symptoms in parents are assessed at two different levels of specificity: the general “*p*-factor” level and the level of internalizing and substance use symptoms (two-factor correlated-factors model). For adult offspring, we assessed three levels of specificity: one general p factor, one two-factor correlated factors model, and one bifactor model. Because of limitations in our data, we were only able to test a one-factor model for minor offspring.

## Methods

### Design

This research uses data collected from two generations (parents and offspring). The original study was based on a prospective cohort design (Leventhal, 1982; Schulsinger et al., 1981) in which abused and neglected children were matched with non-abused and non-neglected children and followed prospectively into adulthood. The individuals from the original study make up the parent generation of the present study and represent individuals with documented histories of childhood abuse and neglect and a demographically matched comparison group of children (Widom, 1989a). These cases of childhood maltreatment (physical and sexual abuse and neglect) were processed during the years 1967 to 1971 in the county juvenile (family) or adult criminal courts of a Midwestern metropolitan area and represent children 11 years of age or less at the time of the incident.

A critical element of the original study design was the identification of a control group of children, matched with the maltreated children on the basis of age, sex, race/ethnicity, and approximate family social class during the time period under study. Matching for approximate family social class was important because it is theoretically plausible that any relationship between child abuse and neglect and subsequent outcomes may be confounded with or explained by social class differences (MacMillan et al., 2001; Widom, 1989b). It was difficult to match exactly for social class because higher-income families could have lived in lower social class neighborhoods and vice versa. The matching procedure used here uses neighborhood schools the children attended and hospitals of birth as a proxy for family social class. When random sampling is not possible, Shadish et al. (2002) recommend using neighborhood and hospital controls to match on variables that are related to outcomes.

Since it is not possible to randomly assign participants to groups, the assumption of equivalence for the groups is an approximation. Official records were checked, and 11 potential comparison group individuals who had an official record of abuse or neglect in their childhood were eliminated. Thus, the control group does not contain any known cases of child abuse or neglect, although the number of participants in the comparison group who were actually abused but not reported is unknown.

### Participants

#### Parent sample

For the purposes of this paper, we use individuals who participated in the first interview during 1989 – 1995 (*N* = 1196) and those who participated in the fourth interview during 2009 – 2020 when offspring were also interviewed. Table 1 shows descriptive statistics for parents with documented histories of childhood maltreatment and matched controls. At the first interview, the sample was 61.5% females, 56.6% white, non-Hispanic, and mean age 28.9 years old. On average, participants had 1.54 children enrolled. Maltreated and control parents did not differ in terms of sex, age, and race, or the number of children enrolled in the study.

**Table 1.** Characteristics of parents with documented histories of childhood maltreatment and matched controls, overall and stratified by sex, and minor and adult offspring

Variable	Maltreated Parents *N* = 238		Control Parents *N* = 205				
Overall	*N*	%	*N*	%	Odds Ratio (95% CI)	*p*	
Female	158	66.4	121	59.0	1.37 (0.93, 2.02)	.110	
White, non-Hispanic	134	56.3	123	60.0	0.86 (0.59, 1.25)	.432	
Teenage parent	87	36.6	69	33.7	1.14 (0.77, 1.68)	.525	
	***M***	**SD**	***M***	**SD**	***T* score**	***p***	**Cohen’s d**
Age at interview 1	28.74	3.70	29.11	3.78	1.04	.301	0.10
Number of children enrolled	1.51	0.50	1.59	0.49	1.73	.085	0.16

Female Parents	Maltreated (*n* = 158)		Controls (*n* = 121)		Odds Ratio (95% CI)		
	*N*	%	*N*	%		*p*	
White, non-Hispanic	85	53.8	68	56.2	0.91 (0.56, 1.46)	.690	
Teenage parent	75	47.5	54	44.6	1.12 (0.70, 1.81)	.637	
	***M***	**SD**	***M***	**SD**	***T* score**	***p***	**Cohen’s d**
Age	28.86	3.60	29.40	3.84	1.21	.225	0.15
Children enrolled	1.51	0.50	1.60	0.49	1.51	.132	0.18

Male Parents	Maltreated (*n* = 80)		Controls (*n* = 84)		Odds Ratio (95% CI)		
	*N*	%	*N*	%		*p*	
White	49	61.3	55	65.5	0.83 (0.44, 1.57)	.575	0.83
Teenage parent	12	15.0	15	17.9	0.81 (0.35, 1.86)	.622	0.81
	***M***	**SD**	***M***	**SD**	**T score**	***p***	**Cohen’s d**
Age	28.51	3.89	28.69	3.67	0.30	.763	0.05
Children enrolled	1.50	0.50	1.57	0.50	0.91	.362	0.14

Minor Offspring	Maltreated (*n* = 70)		Controls (*n* = 87)		Odds Ratio (95% CI)	*p*	
	*N*	%	*N*	%			
Female	31	44.3	46	52.9	0.71 (0.37, 1.33)	.285	
White, non-Hispanic	42	60.0	54	62.1	0.92 (0.48, 1.75)	.792	
	***M***	**SD**	***M***	**SD**	***T* score**	***p***	**Cohen’s d**
Age	13.69	2.82	13.53	2.76	0.35	.726	0.06
Children enrolled	1.63	0.49	1.75	0.44	1.61	.110	0.26

Adult Offspring	Maltreated (*n* = 289)		Controls (*n* = 239)		Odds Ratio (95% CI)	*p*	
	*N*	%	*N*	%			
Female	154	53.3	109	45.6	1.36 (0.97, 1.92)	.079	
White, non-Hispanic	168	58.1	134	56.1	1.09 (0.77, 1.54)	.633	
	***M***	**SD**	***M***	**SD**	***T* score**	***p***	**Cohen’s d**
Age	24.82	4.54	24.88	4.60	0.15	.882	0.01
Children enrolled	1.69	0.47	1.74	0.44	1.40	.163	0.12

*Notes.* SD = standard deviation. As defined by the CDC, teenage parent refers to having a child before the age of 20.

As in any longitudinal study, there is attrition associated with death, refusals, and an inability to locate participants over the waves of the study. To determine whether maltreated participants were more likely to drop out of the study before wave 4 when the offspring were assessed, we examined whether parents who were included differed significantly from parents who did not participate in that interview. Supplementary Table 2 shows that parents who were included in these analyses were significantly more likely to be female than male and to have fewer alcohol abuse and/or dependence disorder symptoms than those who did not participate in this wave of the study.

#### Offspring sample

We collected information about offspring in the context of a study of the intergenerational transmission of child abuse and neglect (Widom et al., 2015). The design required the selection of the firstborn biological child and, if more than one biological child was eligible, a second randomly chosen biological child was also selected. If the firstborn child was not eligible, another biological child who was born within two years of the firstborn was selected. In the few instances where no firstborn was eligible, another biological child would be randomly selected. When biological children were not an option for selection (e.g., none were reported or all were ineligible), a non-biological child was randomly selected. At the time of interview 4, children were excluded if they were under 8 years of age or did not live with or have any contact with the parent before the age of 12. [For details of the offspring selection procedures, see Widom et al. (2015)].

Although 697 children were interviewed, only 685 had parents who also participated in interview 4. For the purpose of this work, we only included the children who had a parent who participated in interview 4. The bottom part of Table 1 shows the characteristics of the offspring sample divided into minor and adult offspring. Overall, the offspring sample was 49.6% female, 58.1% were white, non-Hispanic, and a mean age of 22.3 years (*SD* = 6.34, range = 8 to 38). Like their parents, the offspring of the maltreated and control parents did not differ in terms of sex, race, mean age, or the number of siblings enrolled in the study.

### Procedures

Parents and their offspring were interviewed in person in their homes or other quiet locations of their choice and administered standardized tests. The interviewers were unaware of the purpose of the study and to the inclusion of an abused and/or neglected group. Participants were also unaware of the purpose of the study. Parents had originally been told that they had been selected to participate as part of a large group of individuals who grew up in the late 1960s and early 1970s. To protect the parent’s privacy, interviewers were not permitted to tell children about their parent’s past participation or that we had obtained their contact information from their parent. At the same time, it was important to be able to provide the offspring with enough information for them to make an informed decision about participating. Offspring were told that they were selected because someone in their family had participated in previous rounds of the study and that the purpose of the study was to understand some of the factors that shape how people change as they grow from being a child to a teenager and throughout adulthood. Potential interviewees were assured their responses would be kept confidential, meaning that parents would not see their children’s responses (and vice versa) or even be told whether or not the child had agreed to participate (except for minors who were currently living with the parent in the study). However, parents and offspring were told that there was one special case where an offspring’s responses would not be kept private. Per mandatory reporting laws, participants were told that if we learned that a minor was being harmed or in danger, then we would have to share that information with the appropriate authorities. Institutional Review Board approval was obtained for the procedures involved in this study (current: CUNY Integrated Institutional Review Board, protocol number 2015-0133). Individuals who participated gave written, informed consent. For individuals with limited reading ability, the consent form was presented and explained verbally.

### Measures

#### Parent maltreatment history

Parent history of childhood maltreatment was assessed through review of official records processed during the years 1967 – 1971. Only court-substantiated cases of child physical and sexual abuse and neglect were utilized to avoid any ambiguity as to what constituted child maltreatment. For the current study, a composite measure of childhood maltreatment was used to indicate any physical abuse, sexual abuse, or neglect, and this variable was coded dichotomously (0 = no abuse history, 1 = history of physical, sexual abuse, and/or neglect).

#### Parent psychopathology

Parent psychopathology was assessed during the 1989 – 1995 in-person interviews with participants in young adulthood (Mean age = 29) using the National Institute of Mental Health Diagnostic Interview Schedule – Revised [DIS-III-R; (Robins et al., 1989)]. The DIS-III-R is a standardized psychiatric assessment that yields Diagnostic and Statistical Manual of Mental Disorders (DSM-III-R) diagnoses (American Psychiatric Association, 1987). The DIS-III-R demonstrates adequate reliability (Robins et al., 1981; Vandiver & Sher, 1991). Symptoms from the following DSM-III-R disorders were assessed: Major Depressive Disorder (MDD), Dysthymia, Generalized Anxiety Disorder (GAD), Post-traumatic Stress Disorder (PTSD), Alcohol abuse and/or dependence, and Drug abuse and/or dependence. Continuous variables reflecting the number of lifetime symptoms for each of these disorders were used in the analyses.

#### Offspring psychopathology

Offspring psychopathology was assessed using self-report measures administered during the 2009-2010 in-person interviews, and developmentally appropriate measures were used for minor (under age 18) and adult offspring (18 and older). Minor offspring ranged in age from 8 to 17 (mean age = 13.6, SD = 2.78), and adult offspring ranged in age from 18 to 38 (mean age = 24.9, SD = 4.56).

##### Anxiety

For minor children and adolescent offspring under the age of 18, the Revised Children’s Manifest Anxiety Scale (RCMAS), a 37-item, self-report instrument designed to measure anxiety in children and adolescents, was administered. Each item is rated on a yes/no scale (Yes = *item is descriptive of the subject’s feelings or actions*, No = *item is generally not descriptive*). A total anxiety score was computed based on 28 items. The RCMAS has demonstrated good validity (Reynolds, 1980) and reliability. In this sample, RCMAS scores ranged from 0 to 26 (*M* = 9.89; *SD* = 6.31) and the items had an internal consistency of 0.83.

For adult offspring, the Beck Anxiety Inventory (BAI; (Beck & Steer, 1993)), a 21-item self-report questionnaire assessing symptoms of anxiety, was administered. Each item is rated for symptom severity on a 4-point Likert scale (0 = *not at all*, & 3 = *severely, I could barely stand it*) based on the individual’s experience over the past week. The BAI demonstrates high internal reliability and good factorial and discriminant validity (Kabacoff et al., 1997). In this sample, BAI scores ranged from 0 to 50 (*M* = 10.85; *SD* = 9.0) and items had an internal consistency of 0.89.

##### Depression

For minor offspring under the age of 18, the Children’s Depression Inventory – Short Version (CDI-S; (Kovacs, 2003)), a 10-item self-report questionnaire developed to provide a rapid assessment of symptoms of depression in children, was administered. For each item, respondents are asked to pick one sentence that describes them best for the past two weeks, which is rated for symptom severity on a 3-point Likert scale (e.g., 0 = *I am sad once in a while*, 1 = *I am sad many times*, 2 = *I am sad all the time*). The items are summed to create a total score. The CDI-S demonstrates adequate reliability and validity (Kovacs, 1985). In this minor offspring sample, CDI-S scores ranged from 0 to 12 ((*M* = 1.88; *SD* = 2.35) and items had an internal consistency of 0.72.

For adult offspring (over age 18), the Center for Epidemiologic Studies Depression scale (CES-D; (Radloff, 1977)), a 20-item self-report questionnaire assessing symptoms of depression, was used. Each item is rated for symptom severity on a 4-point Likert scale (0 = *rarely or none of the time [less than a day]*, & 3 = *most or all of the time [5 – 7 days]*) based on the individual’s experience over the past week. The CES-D demonstrates high reliability and validity as a measure of depression (Radloff, 1977). In this adult offspring sample, CES-D scores ranged from 0 to 52 (*M* = 14.39; *SD* = 10.30), and items had an internal consistency of 0.89.

##### Post-traumatic stress disorder (PTSD)

For minor offspring under age 18, the PTSD Index for DSM IV (Rodriguez et al., 2001), a 22-item structured interview, was administered to assess for DSM-IV PTSD symptoms. First, respondents are asked to respond to a series of questions regarding acute (single incident) types of traumatic events and chronic trauma events (criterion A). Part II asks about the frequency of PTSD symptoms during the past month and ever (0 = *not at all*, 1 = *a little of the time*, 2 = *some of the time*, 3 = *much of the time*, 4 = *most of the time)*. If the symptoms were not present in the past month, respondents are asked to indicate if each symptom was ever present in their lifetime (0 = *no*, 1 = *yes*). The items in Part II map directly onto the DSM-IV PTSD criterion B (intrusion), criterion C (avoidance/numbing), and criterion D (arousal). The PTSD Index for DSM-IV demonstrates high validity and reliability (Rodriguez et al., 2001; Roussos et al., 2005; Steinberg et al., 2004). In the minor offspring sample, the yes/no PTSD lifetime symptom variables were summed to represent the total number. Minor offspring lifetime PTSD symptom scores ranged from 0 to 22 (*M* = 9.81; *SD* = 5.94), and the items had an internal consistency of 0.91.

For adult offspring over age 18, a modified version of the Composite International Diagnostic Interview (CIDI) Posttraumatic Stress Disorder (World Health Organization, 1997), a structured diagnostic interview, was administered. The CIDI assesses experience of traumatic events, subjective experience related to the trauma, and lifetime PTSD symptoms (0 = *symptom not present*, 1 = *symptom present*). The CIDI also asks the participant to rate their current level of distress/discomfort related to PTSD symptoms on a scale from 0 = *none* to 4 = *extreme/incapacitating* and has demonstrated acceptable validity and reliability (Peters et al., 1996). In this adult offspring sample, CIDI current symptom scores ranged from 0 to 20 (*M* = 9.81; *SD* = 5.49) and internal consistency of the CIDI was 0.83.

##### Alcohol and drug use

The Wave IV Add Health Pretest Tobacco, Alcohol, Drugs used in the National Longitudinal Study of Adolescent Health (Add Health; (Harris et al., 2003) was administered to assess the use of alcohol and illegal use of prescription drugs and illicit drugs. The Add Health measure is a 57 item, self-report instrument, where reporting the use of legal or illegal drugs prompts further questions about the frequency of use, age of first use, quantity, types of substances, and current use. The response options vary based on the type of questions asked (e.g., yes – no and open-ended responses). For alcohol and drug use, participants were asked a total of 12 questions each. The Add Health measures have demonstrated adequate reliability and validity (Sieving et al., 2001). In the adult offspring sample, Add Health lifetime alcohol use symptom count scores ranged from 0 to 10 (*M* = 0.76; *SD* = 1.85), and Add Health lifetime drug use symptom count scores ranged from 0 to 10 (*M* = 0.50; *SD* = 1.78). Lifetime marijuana use symptom count scores for adult offspring range from 0 to 10 (*M* = 0.84; *SD* = 1.84). In the minor offspring sample, Add Health lifetime alcohol use symptom count scores ranged from 0 to 5 (*M* = 0.08; *SD* = 0.56), and Add Health lifetime drug use symptom count scores ranged from 0 to 1 (*M* = 0.01; *SD* = 0.09). Lifetime marijuana use symptom count scores for minor offspring range from 0 to 7 (*M* = 0.16; *SD* = 0.87).

#### Control variables

Control variables included offspring sex (male = 0, female = 1), offspring race (White, non-Hispanic = 1, all others = 2), and offspring age during the 2009-2010 interviews. Structural equation models did not control for offspring age because they were conducted separately for minor and adult offspring. In addition, because childhood maltreatment has been associated with increased risk for teenage pregnancy and parenting (Madigan et al., 2014), a teenage parent variable was created and included as a control based on the parent’s age at the time their first child was born. This was determined by subtracting the oldest child’s date of birth (DOB) from the parent’s DOB. This information was then coded into a dichotomous variable categorizing individuals who were < 20 years old when they first became parents as 1 = *teenage parent* and individuals who were 20 years or older when they first became parents as 0 = *not a teenage parent*, based on criteria provided by Centers for Disease Control and Prevention (Driscoll et al., 2024).

### Statistical analysis

The first step in the analyses was to compute descriptive statistics for the study variables to compare maltreated and control parents, as well as their offspring. To examine differences in psychiatric symptoms, we conducted independent samples t-tests comparing maltreated and control parents and their offspring. All variables were assessed for normality using Shapiro – Wilk tests and homoscedasticity was assessed using Breusch – Pagan tests. To examine whether parent psychiatric symptoms predicted offspring psychiatric symptoms, we conducted a series of cluster-adjusted linear regressions using Mplus Version 8.11. Parent and offspring symptoms were standardized. Parent symptoms included depression, dysthymia, anxiety, PTSD, alcohol use, and drug use. Adult offspring symptoms included depression, anxiety, PTSD, and alcohol, drug, and marijuana use. Due to the very low rates of substance use reported by minor offspring, minor offspring psychopathology refers only to depression, anxiety, and PTSD. Regressions controlled for offspring age, sex, and race, and were repeated stratified by parent sex. We report p-values and adjusted p-values using the false discovery rate correction due to the number of analyses.

The next step involved using confirmatory factor analysis (CFA) to determine whether these data reflect acceptable factor structures for use in the structural equation models (see Tables 2 and 3). Based on prior literature (Caspi et al., 2014), we tested models reflecting a general p-factor structure as well as models distinguishing between internalizing and substance use domains. For the parents, we tested: (a) a one-factor general psychopathology model, and (b) a two-factor model with correlated internalizing (depression, anxiety, and PTSD) and substance use (alcohol use and drug use for both) dimensions. For the adult offspring, we tested a two-factor model, a one-factor model, and a bifactor model with a general factor and orthogonal internalizing and substance use subfactors. A bifactor model could not be tested for parents due to the limited number of substance use indicators (only alcohol and drug use). CFA results supported the two-factor model for parents and the bifactor model for adult offspring.

**Table 2.** Fit Indices Factor Loadings, and Factor Correlations for Parent Psychopathology Confirmatory Factor Analysis Models

	Parents				
	One-factor model		Two-factor model		
	Model fit	General psychopathology factor (*p*)	Model fit	Internalizing	Substance use
**Statistic**					
Chi-square	123.234***		17.356*		
df	9		8		
CFI	0.880		0.990		
TLI	0.799		0.981		
RMSEA [90% CI]	0.136 [0.115, 0.158]		0.041 [0.013, 0.068]		
**Factor loading**					
Depression		1.000		1.000	
Dysthymia		0.993		0.995	
Anxiety		0.652		0.651	
PTSD		0.507		0.505	
Alcohol		0.308			1.000
Drug		0.350			1.144
**Factor Correlations**					
Internalizing					0.403
Substance Use					

*Note. p* = general psychopathology factor; RMSEA = root mean square error of approximation; CI = confidence interval. Standardized symptom variables are used. **p < .01. ***p < .001

**Table 3.** Fit Indices, Factor Loadings, and Factor Correlations for Adult Offspring Psychopathology Confirmatory Factor Analysis Models

	Adult Offspring								
	Two-factor model			One-factor model		Bifactor model			
	Model fit	Internalizing	Substance Use	Model fit	*p*	Model fit	Internalizing	Substance Use	*p*
**Statistic**									
Chi-square	25.907**			137.581***		3.061			
df	8			9		3			
CFI	0.961			0.720		1.000			
TLI	0.927			0.533		0.999			
RMSEA [90% CI]	0.065 [0.038, 0.094]			0.164 [0.141, 189]		0.006 [0.000, 0.074]			
**Factor loading**									
Depression		1.000			1.000		1.000		1.000
Anxiety		0.785			0.846		0.636		0.797
PTSD		0.437			0.472		0.185		1.134
Alcohol			1.000		0.289			1.000	0.987
Drug			1.465		0.161			2.126	0.640
Marijuana			1.302		0.191			1.590	0.692
**Factor Correlations**									
Internalizing			0.202					0.000	0.000
Substance Use									0.000

*Note.* p = general psychopathology factor/p factor; RMSEA = root mean square error of approximation; CI = confidence interval. Standardized symptom variables are used. **p < .01. ***p < .001

For minor offspring, we were only able to use three variables to measure psychopathology (anxiety, depression, and PTSD). We ran a one-factor CFA model to determine whether an internalizing factor could be used in subsequent analyses for minor offspring. Because this model was saturated, we could not test model fit with CFA to address the underlying structure of the data. Reliability analyses with these three variables yielded a Cronbach’s alpha of 0.82 based on standardized items, indicating good internal consistency within this factor. These results confirmed that a one-factor solution representing internalizing psychopathology was appropriate for minor offspring (see Islam et al., 2022).

After identifying these factors using CFA, we conducted a series of structural equation models (SEM) to test relationships between parent childhood maltreatment history, parent psychopathology, and offspring psychopathology. SEM allows for associations to be simultaneously evaluated rather than estimating multiple independent regressions and also permits estimation of both direct and indirect effects (Bollen, 1987). To account for the nested structure of our data, we conducted our analyses in Mplus and specified clustering by a unique family ID. This approach adjusts standard errors and model fit statistics to account for the non-independence of observations within families, specifically addressing the nesting of multiple offspring within the same parent (Muthén & Muthén, 1998-2017).

In these models, parent childhood maltreatment history was the independent variable, the latent parent internalizing psychopathology and substance use factors were mediators, and dependent variables were offspring latent internalizing psychopathology, and for adult offspring, the substance use factor. Despite CFA results showing that the bifactor model fit best for the adult offspring psychopathology, we opted to use the two-factor model in mediation analyses due to model convergence errors arising with the bifactor model, most likely due to limited sample size. Control variables included teenage parent (included on paths leading to parent psychopathology), and offspring sex and race (included on all paths leading to offspring psychopathology). All of these models were then repeated stratified by parent sex.

We report indirect effects that represent whether parent history of childhood maltreatment predicts offspring psychopathology through the mediating variables (parent psychopathology) in the models. Direct paths were also estimated between parent childhood maltreatment history and offspring outcomes, and between parent psychopathology symptoms and offspring psychopathology symptoms, to explore any influences above and beyond the hypothesized pathways. Maximum likelihood estimation with robust standard errors (MLR) was used due to the non-normality of the data and resulted in the best model fit indices. Model fit was evaluated using the Comparative Fit Index (CFI), Tucker Lewis Index (TLI), and Root Mean Square Error of Approximation (RMSEA), as well as on examining structural paths, their size and statistical significance, following recommendations by Rucker et al. (2011). The values of CFI and TLI > .90, and RMSEA < .08 indicate acceptable model fit (Hu & Bentler, 1998). Full Information Maximum Likelihood (FIML) estimation was used to handle missing data. We used R (version 4.0.3) for descriptive statistics and correlations. Mplus was used for all other analyses.

## Results

### Parents’ history of childhood maltreatment and symptoms of psychiatric disorders

To test the first hypothesis, we examined whether lifetime psychiatric symptoms of parents with documented histories of childhood maltreatment were significantly higher than for controls (see Table 4). These results showed that maltreated parents had a significantly higher number of symptoms of depression, dysthymia, and PTSD, compared to parents without those histories. Maltreated and control parents did not differ in terms of symptoms of generalized anxiety disorder, alcohol abuse/dependence, or drug abuse/dependence.

**Table 4.** Lifetime psychiatric symptoms of maltreated and control parents, overall and stratified by sex, and minor and adult offspring

Variable	Maltreated parents *N* = 238		Control parents *N* = 205				
Overall	Mean	SD	Mean	SD	T score	*p*	Cohen’s d
Depression	3.84	2.75	3.27	2.67	2.21	**.027**	0.21
Dysthymia	2.83	2.16	2.23	1.98	3.05	**.002**	0.29
PTSD	6.26	6.06	4.79	5.58	2.64	**.009**	0.25
GAD	4.61	5.38	3.89	4.78	1.48	.140	0.14
Alcohol	2.37	2.68	2.10	2.50	1.12	.264	0.11
Drug	1.79	2.66	1.57	2.32	0.91	.365	0.09

Female	Maltreated Parents (*n* = 158)		Control Parents (*n* = 121)				
	Mean	SD	Mean	SD	T score	*p*	Cohen’s d
Depression	4.1	2.9	3.6	2.8	1.69	.093	0.20
Dysthymia	3.1	2.3	2.4	2.0	2.58	**.010**	0.31
PTSD	7.2	6.5	5.5	6.0	2.20	**.029**	0.27
GAD	4.9	5.7	4.5	5.2	0.58	.561	0.07
Alcohol	2.0	2.6	1.4	2.2	2.10	**.036**	0.25
Drug	1.5	2.7	1.1	2.1	1.26	.207	0.15

Male	Maltreated Parents (*n* = 80)		Control Parents (*n* = 84)				
	Mean	SD	Mean	SD	T score	*p*	Cohen’s d
Depression	3.3	2.4	2.9	2.5	1.08	.281	0.17
Dysthymia	2.3	1.8	1.9	1.8	1.20	.230	0.19
PTSD	4.4	4.7	3.7	4.8	0.94	.347	0.15
GAD	4.0	4.7	3.0	3.9	1.56	.120	0.24
Alcohol	3.1	2.8	3.1	2.6	0.05	.960	0.01
Drug	2.3	2.6	2.2	2.4	0.34	.735	0.05

Minor Offspring	Maltreated Parents (*N* = 70)		Controls (*N* = 87)				
	Mean	SD	Mean	SD	T score	*p*	Cohen’s d
Depression	2.37	2.79	1.49	1.84	2.36	**.019**	0.38
PTSD	10.04	6.68	9.63	5.34	0.35	.728	0.07
Anxiety	10.78	6.80	9.12	5.80	1.60	.112	0.26
Alcohol use	0.14	0.75	0.04	0.34	0.99	.325	0.17
Drug use	0.00	0.00	0.01	0.11	0.89	.376	0.15
Marijuana use	0.19	0.86	0.14	0.87	0.28	.782	0.05

Adult Offspring	Maltreated Parents (*N* = 289)		Controls (*N* = 239)				
	Mean	SD	Mean	SD	T score	*p*	Cohen’s d
Depression	14.44	9.90	14.32	10.78	0.13	.900	0.01
PTSD	10.15	5.50	9.41	5.46	1.45	.148	0.14
Anxiety	10.58	9.17	11.18	9.18	0.74	.458	0.07
Alcohol use	0.85	1.98	0.66	1.68	1.23	.219	0.11
Drug use	0.44	1.67	0.58	1.90	0.93	.353	0.08
Marijuana use	0.88	1.94	0.80	1.72	0.45	.652	0.04

*Notes*. SD = standard deviation; Psychiatric symptoms represent the number of lifetime DSM-III-R symptoms based on the NIMH Diagnostic Interview Schedule III-R. Depression = major depressive disorder; PTSD = post-traumatic stress disorder; GAD = generalized anxiety disorder; Alcohol and Drug refer to alcohol abuse and/or dependence and drug abuse and/or dependence respectively. CDI = The Children’s Depression Inventory for offspring under 18; CES-D = depression measure for offspring 18 and older; PTSD = post-traumatic stress disorder; PTSD Index = PTSD Index for DSM-IV for offspring under 18; CIDI = Composite International Diagnostic Interview for offspring 18 and older; RCMAS = Revised Children’s Manifest Anxiety Scale for offspring under age 18; BAI = Beck Anxiety Inventory for offspring 18 and older. Offspring depression and anxiety scores represent total current symptom severity. Offspring PTSD, alcohol use, drug use, and marijuana use scores represent the total number of lifetime symptoms.

Table 4 also shows lifetime psychiatric symptoms for male and female maltreated and control parents. Among females, maltreated parents had significantly more dysthymia, PTSD, and alcohol abuse and/or dependence symptoms than control parents, but did not differ significantly in terms of depression, generalized anxiety disorder, or drug abuse and/or dependence symptoms. For males, maltreated and control parents in the sample did not differ significantly on any of the psychiatric disorders assessed here.

### Offspring of parents and psychological symptoms

The bottom of Table 4 shows the psychiatric symptoms of minor and adult offspring of maltreated parents and control parents separately. In contrast to expectations, the only difference between the offspring of maltreated and control parents was that the minor offspring (under 18 years old) of maltreated parents had significantly more symptoms of depression on the CDI than minor offspring of control parents. Adult offspring of maltreated and control parents did not differ on these measures.

### Parent psychopathology and offspring psychopathology

Table 5 shows the extent to which parent psychopathology predicts offspring psychopathology for the sample overall and for male and female parents separately. Overall, parent psychopathology predicted offspring psychopathology. However, different forms of parent psychopathology predicted different forms of offspring psychopathology. Offspring of parents with higher levels of depression and dysthymia had significantly more symptoms of depression, anxiety, PTSD, alcohol use, drug use, and marijuana use. Offspring of parents with higher levels of anxiety had more symptoms of alcohol, drug, and marijuana problems. Offspring of parents with higher levels of symptoms of alcohol abuse and/or dependence had offspring with more symptoms of PTSD and alcohol use and parents with more drug abuse and/dependence symptoms had offspring with more symptoms of alcohol use and marijuana use.

**Table 5.** Cluster-adjusted linear regressions testing whether parent psychiatric symptoms predict offspring psychiatric symptoms overall and stratified for male and female parents separately

	Offspring																							
	Depression				Anxiety				PTSD				Alcohol (Adult only)				Drug (Adult only)				Marijuana (Adult only)			
	β	SE	*p*	adj. *p*	β	SE	*p*	adj. *p*	*β*	SE	*p*	adj. *p*	*β*	SE	*p*	adj. *p*	*β*	SE	*p*	adj. *p*	*β*	SE	*p*	adj. *p*
**Parent**																								
DEP	0.12	0.04	**.003**	**.011**	0.12	0.04	**.004**	**.011**	0.10	0.04	**.012**	.**024**	0.13	0.04	**.002**	**.010**	0.16	0.05	**.003**	**.011**	0.18	0.05	**< .001**	**.009**
DYS	0.12	0.04	**.004**	**.011**	0.12	0.04	**.007**	**.015**	0.14	0.04	**.002**	**.010**	0.15	0.05	**.002**	**.010**	0.18	0.06	**.005**	**.012**	0.18	0.06	**< .001**	**.009**
GAD	0.03	0.04	.437	.524	0.08	0.04	.064	.100	0.05	0.04	.215	.276	0.14	0.05	**.005**	**.012**	0.17	0.06	**.004**	**.011**	0.23	0.06	**< .001**	**.009**
PTSD	0.03	0.04	.478	.538	0.08	0.04	.060	.100	0.03	0.04	.473	.538	0.01	0.05	.872	.872	−0.02	0.05	.661	.700	0.01	0.05	.849	.872
ALC	0.02	0.04	.626	.683	0.04	0.04	.334	.414	0.11	0.05	**.024**	**.045**	0.19	0.05	**< .001**	**.009**	0.08	0.06	.169	.234	0.06	0.05	.159	.229
DRG	0.06	0.04	.124	.186	0.05	0.04	.207	.276	0.08	0.05	.062	.100	0.16	0.05	**.003**	**.011**	0.12	0.06	.053	.095	0.15	0.05	**.007**	**.015**
**Female parents**																								
DEP	0.08	0.05	.132	.183	0.11	0.05	**.024**	.062	0.11	0.05	**.031**	.070	0.17	0.03	**.006**	**.048**	0.13	0.07	**.049**	.093	0.24	0.07	**< .001**	**.012**
DYS	0.11	0.05	**.048**	.093	0.13	0.05	**.014**	**.046**	0.15	0.05	**.005**	**.030**	0.18	0.05	**< .001**	**.030**	0.17	0.08	**.026**	.062	0.23	0.07	**.002**	**.018**
GAD	0.01	0.05	.877	.902	0.09	0.05	.089	.139	0.04	0.05	.420	.488	0.17	0.06	**.005**	**.030**	0.16	0.07	**.024**	.062	0.29	0.07	**< .001**	**.012**
PTSD	0.02	0.05	.683	.768	0.07	0.05	.150	.193	0.02	0.05	.754	.823	−0.01	0.05	.800	.847	−0.10	0.06	.106	.159	−0.01	0.07	.919	.919
ALC	0.08	0.06	.217	.260	0.12	0.05	**.042**	.089	0.16	0.05	**.008**	**.036**	0.15	0.06	**.010**	**.040**	0.09	0.06	.131	.183	0.08	0.06	.193	.240
DRG	0.10	0.05	.070	.115	0.10	0.05	.060	.105	0.09	0.06	.143	.191	0.17	0.07	**.014**	**.046**	0.13	0.07	.061	.105	0.19	0.08	**.016**	**.048**
**Male parents**																								
DEP	0.18	0.06	**.002**	**.036**	0.10	0.07	.173	.506	0.09	0.07	.207	.506	0.12	0.08	.135	.486	0.19	0.10	.073	.438	0.04	0.06	.464	.690
DYS	0.14	0.06	**.029**	.348	0.05	0.08	.522	.716	0.10	0.08	.176	.506	0.05	0.08	.537	.716	0.18	0.11	.117	.468	0.06	0.08	.449	.690
GAD	0.08	0.07	.225	.506	0.03	0.07	.646	.724	0.08	0.08	.286	.572	0.06	0.08	.477	.690	0.19	0.12	.096	.468	0.05	0.07	.477	.690
PTSD	0.02	0.07	.790	.813	0.04	0.08	.651	.724	0.06	0.09	.479	.690	0.11	0.09	.213	.506	0.22	0.11	.051	.396	0.03	0.07	.630	.724
ALC	−0.03	0.06	.678	.724	0.03	0.06	.675	.724	0.03	0.07	.684	.724	0.24	0.07	**.001**	**.036**	0.08	0.08	.362	.652	0.06	0.06	.333	.631
DRG	0.03	0.06	.663	.724	0.00	0.06	.944	.944	0.09	0.07	.190	.506	0.17	0.09	.055	.396	0.13	0.11	.261	.553	0.10	0.06	.117	.468

*Note*. Est. = Estimate; SE = standard error; β = standardized regression coefficient; Adj. p = p-value adjusted using the false discovery rate (FDR) correction; DEP = depression; DYS = dysthymia; GAD = generalized anxiety disorder; PTSD = posttraumatic stress disorder; ALC = alcohol abuse and/or dependence; DRG = drug abuse and/or dependence. Parent psychiatric symptoms represent the number of lifetime symptoms. Offspring and parent variables were standardized. Robust standard errors are reported. Analyses control for offsprings’ age, sex, and race. Bolded values indicate significance.

For female parents, those with more symptoms of depression and dysthymia had offspring with more symptoms of anxiety, PTSD, alcohol use, drug use, and marijuana use. In addition, offspring with female parents with higher levels of anxiety symptoms had higher levels of alcohol, drug, and marijuana problems. Female parents with alcohol problems had offspring with higher levels of anxiety, PTSD, and alcohol problems, whereas female parents with drug problems had offspring with more alcohol and marijuana problems.

For male parents, those with more depression symptoms had offspring with more depression symptoms. Similarly, male parents with more alcohol abuse and/or dependence symptoms had offspring with more alcohol symptoms.

### Does parent psychopathology mediate the relationship between parental maltreatment history and offspring psychopathology?

#### Adult offspring

**Figure 1.**
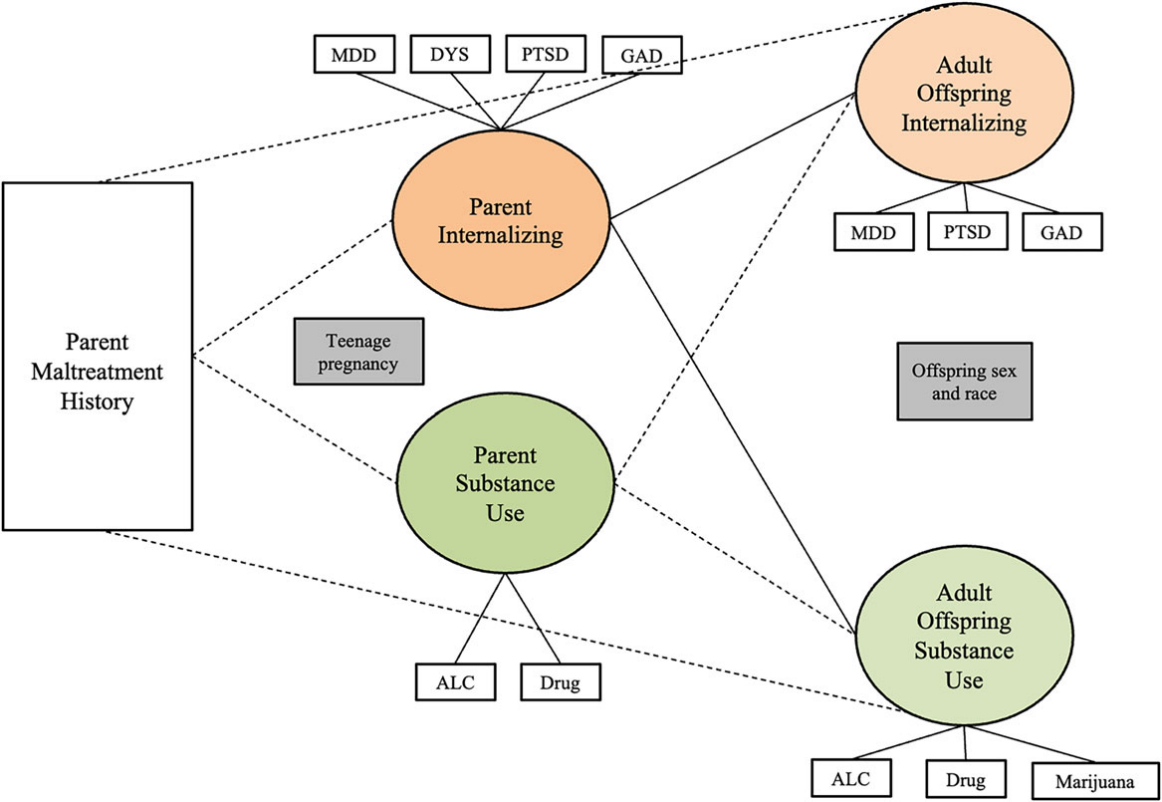
Results of structural equation model testing whether two-factor parent psychopathology construct (internalizing and substance use) mediates the relationship between childhood maltreatment and adult offspring internalizing psychopathology and substance use. *Note.* Rectangles represent observed variables; ovals represent latent variables on which psychopathology scores have been regressed. Teenage pregnancy was controlled for on the paths leading to parent psychopathology. Offspring sex and race were controlled for on paths leading to the outcome variables. Dotted lines represent non-significant paths. Solid lines represent paths with significant coefficients.

**Figure 2.**
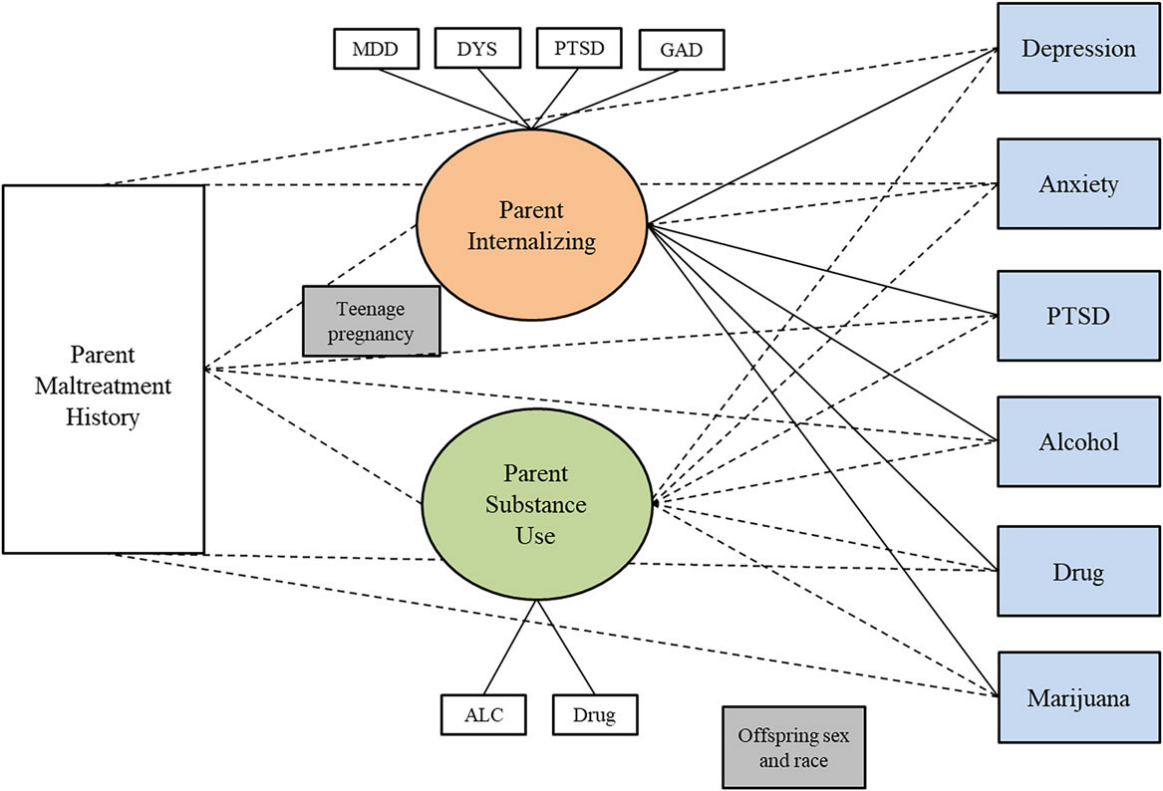
Results of structural equation model testing whether the two-factor parent psychopathology construct (internalizing and substance use) mediates the relationship between childhood maltreatment and adult offspring individual symptoms. *Note.* Rectangles represent observed variables; ovals represent latent variables on which psychopathology scores have been regressed. Teenage pregnancy was controlled for on the paths leading to parent psychopathology. Offspring sex and race were controlled for on paths leading to the outcome variables. Dotted lines represent non-significant paths. Solid lines represent paths with significant coefficients.

**Figure 3.**
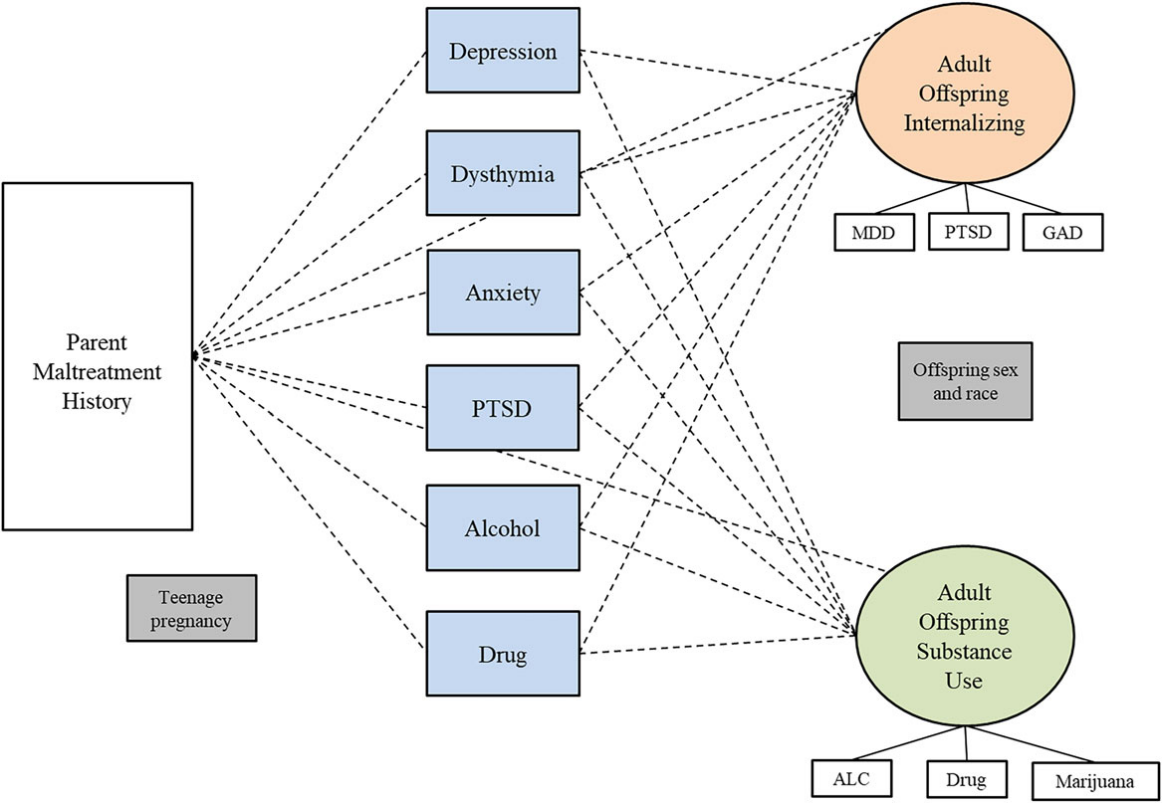
Results of structural equation model testing whether parent individual symptoms mediate the relationship between childhood maltreatment and two-factor adult offspring psychopathology construct (internalizing and substance use). *Note.* Rectangles represent observed variables; ovals represent latent variables on which psychopathology scores have been regressed. Teenage pregnancy was controlled for on the paths leading to parent psychopathology. Offspring sex and race were controlled for on paths leading to the outcome variables. Model fit indices were poor; therefore, we did not interpret the *p*-values for each path.

Table 6 shows the results of SEM models testing whether the two-factor parent psychopathology construct (internalizing and substance use) mediates the relationship between parent’s childhood maltreatment history and adult offspring psychopathology. For the adult offspring sample, overall and for male and female parents separately, the indirect paths were not significant, indicating no evidence of mediation, in any of the models. Figures 1, 2, and 3 show the results of three separate SEM models testing whether the relationship between a parent’s history of childhood maltreatment and adult offspring psychopathology is mediated by parent psychopathology. Figure 1 shows that parent internalizing psychopathology predicts offspring internalizing psychopathology and substance use, but no evidence of mediation. Figure 2 shows that parent internalizing psychopathology predicts all adult offspring individual symptoms, whereas Figure 3 shows that there were no significant paths from individual parent psychopathology symptoms to the two-factor adult offspring psychopathology construct (internalizing and substance use).

**Table 6.** Results of SEM model testing whether two-factor parent psychopathology (internalizing and substance use) mediates the relationship between childhood maltreatment and adult offspring internalizing psychopathology and substance use

	Est.	SE	95% CI	*p*	χ ^2^	CFI	TLI	RMSEA
***Total Sample***								
Total effect	0.02	0.12	−0.19, 0.22	.894	203.908***	0.931	0.910	0.050
Parent CM -> Adult offspring internalizing	−0.02	0.09	−0.17, 0.13	.832				
Parent CM -> Adult offspring substance use	−0.01	0.06	−0.11, 0.08	.823				
Parent internalizing -> Adult offspring internalizing	0.11	0.05	0.02, 0.20	**.036**				
Parent internalizing -> Adult offspring substance use	0.14	0.05	0.06, 0.22	**.006**				
Parent substance -> Adult offspring internalizing	−0.05	0.07	−0.16, 0.07	.485				
Parent substance -> Adult offspring substance use	0.09	0.08	−0.04, 0.22	.243				
Parent CM -> Parent internalizing	0.17	0.11	−0.01, 0.34	.118				
Parent CM -> Parent substance	0.15	0.14	−0.08, 0.38	.289				
Parent CM -> Parent internalizing -> Adult offspring internalizing	0.02	0.02	−0.01, 0.04	.213				
Parent CM -> Parent internalizing -> Adult offspring substance	0.02	0.02	−0.00, 0.05	.157				
Parent CM -> Parent substance -> Adult offspring internalizing	−0.01	0.01	−0.03, 0.02	.613				
Parent CM -> Parent substance -> Adult offspring substance	0.01	0.01	−0.01, 0.03	.241				
Total indirect effect	0.05	0.03	−0.01, 0.10	.147				
***Female Parents***								
Total effect	−0.04	0.16	−0.31, 0.22	.788	210.065***	0.895	0.863	0.064
Parent CM -> Adult offspring internalizing	−0.12	0.12	−0.32, 0.07	.294				
Parent CM -> Adult offspring substance use	0.02	0.08	−0.11, 0.15	.807				
Parent internalizing -> Adult offspring internalizing	0.07	0.07	−0.05, 0.19	.309				
Parent internalizing -> Adult offspring substance use	0.16	0.06	0.05, 0.26	**.014**				
Parent substance -> Adult offspring internalizing	0.02	0.10	−0.14, 0.18	.842				
Parent substance -> Adult offspring substance use	0.07	0.11	−0.11, 0.25	.499				
Parent CM -> Parent internalizing	0.16	0.14	−0.07, 0.38	.246				
Parent CM -> Parent substance	0.26	0.15	0.01, 0.50	.087				
Parent CM -> Parent internalizing -> Adult offspring internalizing	0.01	0.02	−0.02, 0.04	.463				
Parent CM -> Parent internalizing -> Adult offspring substance	0.03	0.02	−0.01, 0.06	.288				
Parent CM -> Parent substance -> Adult offspring internalizing	0.01	0.02	−0.03, 0.04	.835				
Parent CM -> Parent substance -> Adult offspring substance	0.02	0.02	−0.02, 0.06	.443				
Total indirect effect	0.06	0.05	−0.02, 0.15	.236				
***Male Parents***								
Total effect	0.22	0.21	−0.13, 0.57	.298	185.869***	0.835	0.783	0.079
Parent CM -> Adult offspring internalizing	0.30	0.18	0.01, 0.59	.090				
Parent CM -> Adult offspring substance use	−0.11	0.10	−0.27, 0.05	.253				
Parent internalizing -> Adult offspring internalizing	0.15	0.09	0.00, 0.31	.096				
Parent internalizing -> Adult offspring substance use	0.07	0.12	−0.13, 0.27	.553				
Parent substance -> Adult offspring internalizing	−0.10	0.10	−0.27, 0.06	.290				
Parent substance -> Adult offspring substance use	0.15	0.48	−0.64, 0.95	.751				
Parent CM -> Parent internalizing	0.12	0.18	−0.18, 0.42	.504				
Parent CM -> Parent substance	0.09	0.20	−0.23, 0.41	.648				
Parent CM -> Parent internalizing -> Adult offspring internalizing	0.02	0.03	−0.03, 0.07	.509				
Parent CM -> Parent internalizing -> Adult offspring substance	0.01	0.02	−0.02, 0.04	.631				
Parent CM -> Parent substance -> Adult offspring internalizing	−0.01	0.02	−0.05, 0.03	.682				
Parent CM -> Parent substance -> Adult offspring substance	0.01	0.05	−0.07, 0.10	.783				
Total indirect effect	0.03	0.07	−0.09, 0.15	.652				

*Notes*. Results from SEM models testing for mediation with parent two-factor model s and adult offspring two-factor models. SE = standard error; CI = confidence interval; χ ^2^ = critical ratio chi square; CFI = comparative fit index; TLI = Tucker–Lewis index; RMSEA = root mean square error of approximation; CM = childhood maltreatment. Offspring and parent psychiatric symptom variables are all standardized. Offspring anxiety and depression represent current symptoms. Offspring PTSD, alcohol use, drug use, and marijuana use represent lifetime symptoms. All analyses controlled for offsprings’ sex and race on the offspring symptom constructs. Being a teenage parent (first child was born when the parent was less than 20 years old) is controlled for on the paths from parent maltreatment to parent symptoms. ***p < .001

#### Minor offspring

**Figure 4.**
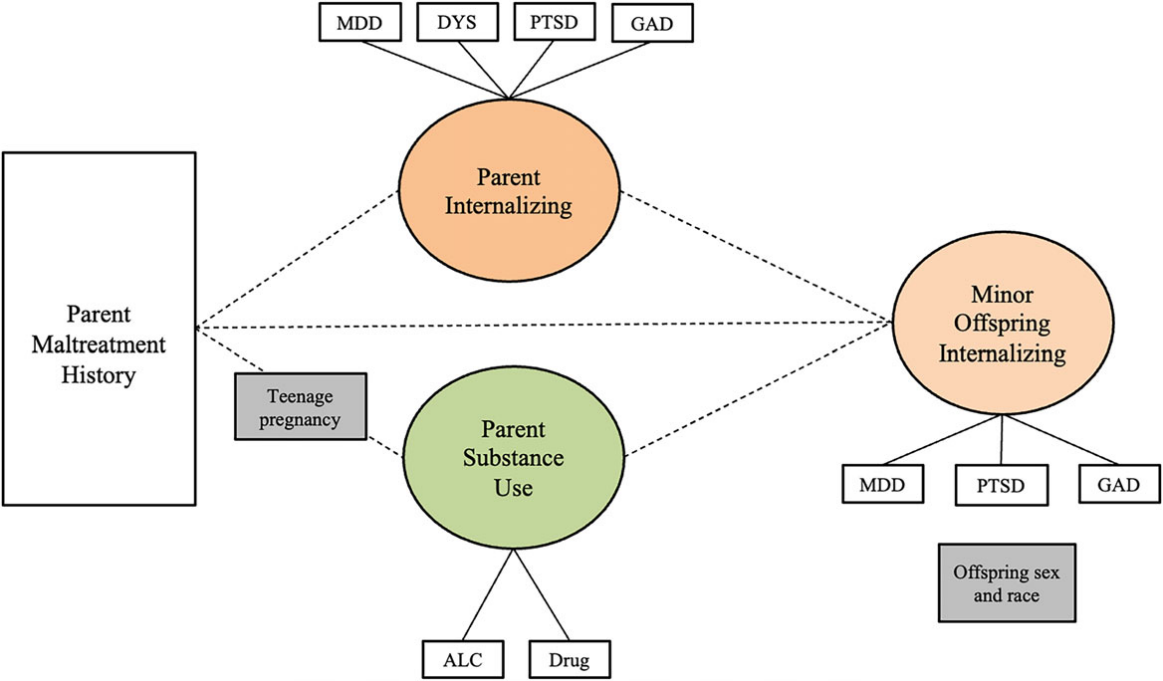
Results of structural equation models testing whether the two-factor parent psychopathology construct (internalizing and substance use) mediates the relationship between childhood maltreatment and minor offspring one-factor internalizing psychopathology construct. *Note.* Rectangles represent observed variables; ovals represent latent variables on which psychopathology scores have been regressed. Teenage pregnancy was controlled for on the paths leading to parent psychopathology. Offspring sex and race were controlled for on paths leading to the outcome variables. Dotted lines represent non-significant paths. Solid lines represent paths with significant coefficients.

**Figure 5.**
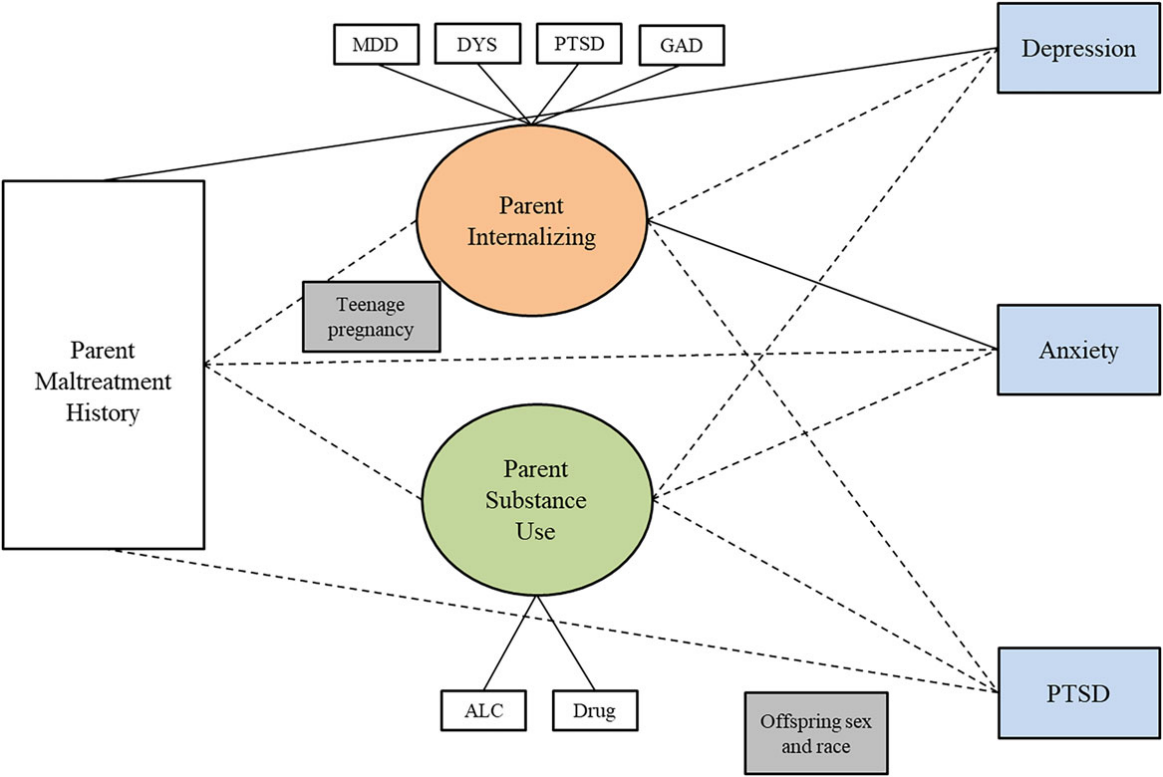
Results of structural equation model testing whether the two-factor parent psychopathology construct (internalizing and substance use) mediates the relationship between childhood maltreatment and minor offspring individual symptoms Minor. *Note.* Rectangles represent observed variables; ovals represent latent variables on which psychopathology scores have been regressed. Teenage pregnancy was controlled for on the paths leading to parent psychopathology. Offspring sex and race were controlled for on paths leading to the outcome variables. Dotted lines represent non-significant paths. Solid lines represent paths with significant coefficients.

**Figure 6.**
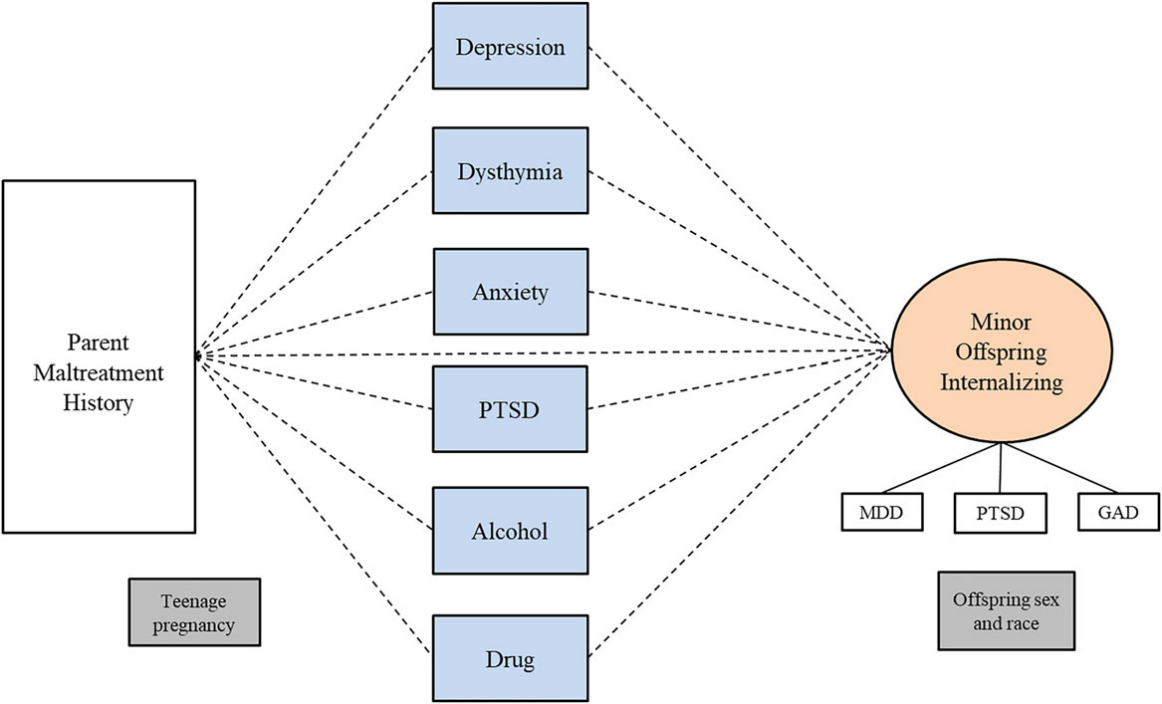
Results of structural equation models testing whether parent individual symptoms mediate the relationship between childhood maltreatment and minor offspring internalizing psychopathology. *Note.* Rectangles represent observed variables; ovals represent latent variables on which psychopathology scores have been regressed. Teenage pregnancy was controlled for on the paths leading to parent psychopathology. Offspring sex and race were controlled for on paths leading to the outcome variables. Model fit indices were poor; therefore, we did not interpret the p-values for each path.

Table 7 shows the results of a SEM model testing whether the two-factor parent psychopathology construct mediates the relationship between a parent childhood maltreatment history and the minor offspring internalizing factor. For the minor sample overall and for female and male parents, none of the indirect paths were significant, indicating no evidence of mediation. Figures 4, 5, and 6 show the results of SEM models testing whether different models show mediation. Figure 4 shows the results of a model testing whether the two-factor parent psychopathology mediates the relationship between parent childhood maltreatment and minor offspring internalizing construct and reveals no significant paths. Figure 5 shows the results of testing whether the two-factor parent psychopathology construct mediates the relationship between parent childhood maltreatment and minor offspring individual symptoms. Figure 5 shows that there is a significant direct path from parent childhood maltreatment to minor offspring depression and another significant direct path from parent internalizing to minor offspring anxiety, but no evidence of mediation. Finally, Figure 6 shows that there are no significant paths in the model testing whether parent individual symptoms predict minor offspring internalizing.

**Table 7.** Results of structural equation models testing whether parent two-factor psychopathology (internalizing and substance use) mediates the relationship between childhood maltreatment and minor offspring internalizing psychopathology

	Est.	SE	95% CI	*p*	χ ^2^	CFI	TLI	RMSEA
***Total Sample***								
Total effect	0.28	0.15	0.04, 0.53	.058	117.301***	0.895	0.860	0.086
Parent CM -> Minor offspring internalizing	0.26	0.14	0.03, 0.50	.065				
Parent internalizing -> Minor offspring internalizing	0.16	0.09	0.02, 0.30	.070				
Parent substance -> Minor offspring internalizing	0.29	0.18	0.00, 0.57	.100				
Parent CM -> Parent internalizing	0.30	0.16	0.03, 0.56	.067				
Parent CM -> Parent substance	−0.10	0.18	−0.40, 0.20	.572				
Parent CM -> Parent internalizing -> Minor offspring internalizing	0.05	0.04	−0.02, 0.11	.215				
Parent CM -> Parent substance -> Minor offspring internalizing	−0.03	0.05	−0.11, 0.05	.535				
Total indirect effect	0.02	0.07	−0.10, 0.13	.797				
***Female Parents***								
Total effect	0.22	0.23	−0.16, 0.59	.347	90.787**	0.893	0.857	0.089
Parent CM -> Minor offspring internalizing	0.16	0.22	−0.20, 0.52	.473				
Parent internalizing -> Minor offspring internalizing	0.15	0.13	−0.07, 0.37	.262				
Parent substance -> Minor offspring internalizing	0.52	0.20	0.20, 0.85	**.009**				
Parent CM -> Parent internalizing	0.38	0.24	−0.02, 0.78	.120				
Parent CM -> Parent substance	0.01	0.24	−0.39, 0.40	.979				
Parent CM -> Parent internalizing -> Minor offspring internalizing	0.06	0.06	−0.05, 0.16	.381				
Parent CM -> Parent substance -> Minor offspring internalizing	0.00	0.13	−0.20, 0.21	.979				
Total indirect effect	0.06	0.16	−0.20, 0.32	.708				
***Male Parents***								
Total effect	0.34	0.17	0.06, 0.63	**.048**	87.391**	0.879	0.839	0.093
Parent CM -> Minor offspring internalizing	0.31	0.17	0.03, 0.59	.068				
Parent internalizing -> Minor offspring internalizing	0.13	0.08	−0.00, 0.26	.106				
Parent substance -> Minor offspring internalizing	−0.02	0.08	−0.15, 0.11	.806				
Parent CM -> Parent internalizing	0.21	0.23	−0.16, 0.59	.353				
Parent CM -> Parent substance	−0.31	0.23	−0.68, 0.07	.182				
Parent CM -> Parent internalizing -> Minor offspring internalizing	0.03	0.03	−0.03, 0.08	.414				
Parent CM -> Parent substance -> Minor offspring internalizing	0.01	0.02	−0.03, 0.04	.791				
Total indirect effect	0.03	0.04	−0.03, 0.10	.374				

*Notes*. Results from SEM models testing for mediation with parent two-factor models and minor offspring one-factor models. SE = standard error; CI = confidence interval; χ^2^ = critical ratio chi square; CFI = comparative fit index; TLI = Tucker – Lewis index; RMSEA = root mean square error of approximation; CM = childhood maltreatment. Offspring and parent psychiatric symptom variables are all standardized. Offspring anxiety and depression represent current symptoms. Offspring PTSD represent lifetime symptoms. All analyses controlled for offsprings’ sex and race on the offspring symptom constructs. Being a teenage parent (first child was born when the parent was less than 20 years old) is controlled for on the paths from parent maltreatment to parent symptoms.

**p < .01. ***p < .001

## Discussion

The current study sought to determine whether parent psychopathology represents one of the mechanisms by which a parent history of childhood maltreatment increases risk for mental health problems in their offspring. To examine this question, we used data from a prospective cohort design study of children with documented cases of maltreatment and a demographically matched control group of children who were followed up into adulthood. We then assessed the extent of mental health symptoms in a sample of their offspring and tested for mediation.

As hypothesized, these results showed that parents with histories of childhood maltreatment had more symptoms of depression, dysthymia, and PTSD, compared to parents without those histories. Contrary to our hypotheses, maltreated and control parents overall did not differ in terms of symptoms of generalized anxiety disorder, alcohol abuse/dependence, or drug abuse/dependence. However, female maltreated parents reported significantly more symptoms of alcohol abuse and/or dependence than control female parents. These findings about alcohol problems are consistent with earlier findings where it was only the females who had been abused or neglected as children who showed increased risk, not the males (Widom et al., 1995). In addition, the attrition analyses indicated that the parents who participated in this wave of the study with their offspring had fewer alcohol abuse and/or dependence symptoms than those who did not participate in this wave of the study and this may have contributed to the lack of overall differences for alcohol symptoms in maltreated parents.

Our comparisons of the extent to which parents with histories of childhood maltreatment had offspring with psychiatric symptoms revealed that offspring of maltreated parents had more symptoms of only one specific disorder – depression – compared to offspring of matched controls, and this difference was only found for minor offspring. This result was contrary to what we had expected in terms of the effect of parent maltreatment on offspring mental health. Although surprising, there are several possible explanations for why this difference might only appear in minor offspring, and not in adult offspring. From a developmental perspective, the minor offspring, who ranged in age from 8 to 17, were in a sensitive period for the development of psychopathology, as many psychiatric disorders originate during childhood and adolescence (Kessler et al., 2005; Merikangas et al., 2010) and the risk for psychopathology is at its highest in adolescence (Fairchild, 2011). Adult offspring mental health may have improved over time, and it is possible that their symptoms subsided by the time they reached adulthood. Adult offspring may also have had more time than minor offspring to access treatment resources (e.g., therapy, medication, self-help resources). In one study, adult offspring of depressed parents used more resources for help with mental health problems in their lifetime and were more likely to have obtained help from a mental health professional in the past year, compared to controls (Timko et al., 2008). Another possibility is that the adult offspring had moved away from their parents and this distance led to changes in symptomatology (Thomeer & Reczek, 2019). Lastly, it is possible that these differences are due to the different measures used to assess psychopathology in minor versus adult offspring. While we argued for the need to consider developmental differences in symptoms in childhood, preadolescence, adolescence, and later adolescence, we were not able to consider these potential differences and were constrained to consideration of only one category (ages 8 – 17).

These results show that parent psychopathology predicted offspring psychopathology. Specifically, offspring of parents with depression and dysthymia had more symptoms of depression, anxiety, PTSD, alcohol use, drug use, and marijuana use – which may suggest that parental depressive symptoms are conferred across generations but nonspecifically. However, offspring symptoms such as dysthymia were not assessed in the current study, and thus it was not possible to directly compare parent psychopathology with offspring psychopathology in every case.

Offspring of parents with more lifetime symptoms of anxiety had more symptoms of alcohol, drug, and marijuana problems, suggesting that parental anxiety may be conferred across generations according to a pattern that is more consistent with heterotypic transmission (e.g., from parent anxiety to offspring substance use). Offspring of parents with more alcohol problems had more symptoms of PTSD and alcohol use. Offspring of parents with more drug symptoms had offspring with more symptoms of alcohol use and marijuana use. These findings regarding the risk of alcohol and drug problems for offspring of parents with alcohol and drug problems are consistent with prior literature that suggests that genetic or hereditary factors contribute to the transmission of alcohol and substance use problems within families (Biederman et al., 2000; Kendler et al., 2024; Merikangas et al., 1998).

Our findings revealed that parent internalizing psychopathology, but not externalizing, substance use, or individual symptoms, most consistently predicted offspring psychopathology, including adult offspring internalizing and substance use, as well as the individual symptoms of depression, anxiety, PTSD, alcohol use, drug use, and marijuana use. However, in contrast to reports in prior literature, there was no evidence of parent psychopathology mediating the relationship between parental childhood maltreatment history and offspring psychopathology.

Thus, contrary to our hypotheses, we did not find evidence that parent psychopathology mediates the relationship between parent childhood maltreatment history and offspring psychopathology in any of the models tested. While these findings were surprising, the present study differs from previous research in a number of ways. First, many of the studies focused on specific disorders or broad indicators of psychological distress. This study examined the effect of a broader range of parent psychopathology as potential mediators and offspring psychological outcomes, rather than focusing on specific disorders only. Based on prior literature (Caspi et al., 2014), we tested models reflecting a general p-factor structure as well as models distinguishing between internalizing and substance use domains and examined multiple domains of parent and offspring psychopathology as latent factors and as individual symptoms. Second, previous studies have relied heavily on retrospective reports of childhood maltreatment to determine parents’ maltreatment history. In the current study, we used documented cases of childhood maltreatment to determine parent maltreatment status. This distinction is important because there is increasing evidence that individuals identified through retrospective reports differ from those whose maltreatment was ascertained based on objective or documented cases. Third, most of the existing research is based only on mothers’ characteristics in relation to offspring psychopathology, whereas the current research has examined both mothers’ and fathers’ maltreatment histories and psychopathology. Fourth, in this study, offspring were assessed directly rather than using parents or teachers to describe characteristics of the offspring. As noted earlier, parent ratings of their offspring may be biased by the parents’ own psychopathology at the time of the assessment (Maoz et al., 2014; Najman et al., 2000; Zhang & Ji, 2024). In some work that used reports of both parent and offspring to assess offspring outcomes, mediation was found for parent psychopathology, but *only* when using parents’ reports, and not when using offspring self-reports. Fifth, studies have assessed offspring in early and middle childhood (Marceau et al., 2022) when symptoms may not have appeared. The present study examined symptoms in young adulthood and reflect lifetime psychopathology and thus permit an evaluation of a longer time period for the offspring. Finally, differences in the study designs (longitudinal versus cross-sectional) may help explain differences in the current findings and those of earlier studies, since cross-sectional designs only present a snapshot of the person’s situation at that point in time.

We did not make predictions about the extent to which male and female parents’ histories of childhood maltreatment and subsequent consequences might have predicted offspring psychopathology in different ways. The unique advantage of this study is the fact that we included male parents in this assessment, not focusing only on female parents. Our results indicated that female parents’ psychiatric problems predicted their offspring’s psychopathology (depression, anxiety, PTSD, alcohol and drug use). For the male parents in the study, these results show that male parents’ symptoms predicted their offsprings’ depression and alcohol problems, despite the fact that the male parent sample was much smaller than the female sample. These new findings suggest that efforts need to be made to better understand the role of male parents when cases of maltreatment are identified. Future studies may benefit from investigating possible gender-specific effects among offspring (e.g., such as paternal mental health affecting sons and/or maternal mental health affecting daughters).

Finally, the fact that parent PTSD symptoms did not predict offspring psychopathology or act as a mediator in our study may be consistent with evidence that PTSD can actually be protective, and specifically in the context of parenting. For example, some studies have found parents with PTSD symptoms to be less likely to engage in abuse of their own children (Pears & Capaldi, 2001). Thus, it is possible that parents’ PTSD symptoms provided a buffering effect for maltreated parents in breaking the cycle of maltreatment (Islam et al., 2022). A more recent study also found that mothers with PTSD symptoms engaged in more responsive parenting, which helped buffer the negative effects of their symptoms on their children (Greene et al., 2020). However, it is important to note that the present analyses do not directly establish that offspring were exposed to their parent’s psychopathology during their own childhood. Parent psychopathology represents the parents’ lifetime symptom histories rather than the onset or recency of the symptoms. Therefore, these results should be interpreted as reflecting the intergenerational association with parent history of psychopathology rather than with direct exposure to active symptoms.

## Limitations

Although this study has many advantages, some limitations should be noted. First, these findings are based on cases of childhood abuse and neglect drawn from official court records and most likely represent the most extreme cases processed in the system. Second, cases that came to the attention of the courts are skewed toward the lower end of the socio-economic spectrum and, therefore, these results cannot be generalized to abused and neglected children who grew up in middle- or upper-class homes. Third, the cases included here represented those that occurred in the late 1960s and early 1970s in the Midwest part of the United States and might differ from cases currently being processed by child protection agencies. However, these cases are comparable in demographic characteristics to the kinds of cases being processed currently by child protection services across the country. Fourth, parents’ psychopathology was measured during the 1989-1995 interviews, when the parents were approximately 29 years old. It is possible that parent psychiatric symptoms might have dissipated over time. It is also possible that the offspring might have been exposed to a parent who had psychiatric symptoms at some point in their lifetime, but not necessarily directly exposed to their parent’s symptoms. Fifth, due to limited sample size, our minor offspring sample represented a wide developmental range (8 – 17 years). Childhood, preadolescence, and adolescence represent distinct developmental periods, and by collapsing these periods into a single group, we may have obscured more specific developmental variation in the expression of symptoms. Sixth, many factors likely play a role in the relationship between parental childhood maltreatment and offspring psychopathology that were not examined here. The current study was not able to assess the influence of different types of abuse (physical abuse, sexual abuse, or neglect), abuse severity, or the experience of multiple types of abuse, which may be important factors to include in future research. Finally, the size of some of the groups compared were relatively small and might have limited our power and ability to detect clinically significant differences.

## Implications and conclusions

The results of the present study contribute to understanding the long-term and intergenerational effects of childhood maltreatment and extends previous research in several ways. First, the current study included both female and male parents in this assessment of parent childhood abuse history on offspring psychopathology. Second, this study examined the effect of a broader range of parent psychopathology potential mediators and offspring psychological outcomes, rather than focusing on specific disorders only. Third, we found that only minor, and not adult, offspring of maltreated parents had higher levels of psychopathology relative to minor offspring of control parents. These findings suggest that the impact of parent childhood maltreatment on offspring psychopathology may vary across development and may differentially influence child risk over time. Thus, efforts to prevent adverse intergenerational effects of a parental childhood maltreatment history should start in early life, as these symptoms may be most significant in childhood. In mental health settings, regular screening for abuse history in parents should be conducted to identify parents and children at risk for mental health problems. This screening may be particularly important to conduct among parents seeking services for their child’s emotional and/or behavioral problems. Disentangling the consequences of trauma on parents, children, and families is not an easy or straightforward task. The results of this longitudinal study of parents with documented histories of childhood maltreatment reveal a complex picture of possible pathways linking parent childhood abuse and neglect to offspring mental health and call attention to parent psychopathology variables – as well as additional factors not included in the present study – as important mediators explaining this relationship. It is likely that a combination of risk and protective factors contributes to intergenerational patterns of trauma and mental health problems.

## Supporting information

10.1017/S0954579425101120.sm001 Young et al. supplementary material Young et al. supplementary material

## Data Availability

The data reported in the current article are not publicly available because they contain sensitive information that could compromise research participant privacy and confidentiality. We cannot provide individual level data from this project because our confidentiality agreement with the participants in this study precludes this. The data are available on request from the corresponding author [CSW] by qualified scientists. Requests require a concept paper describing the purpose of data access, ethical approval at the applicant’s university in writing, and provision for secure data access.

## Supplementary material

The supplementary material for this article can be found at https://doi.org/10.1017/S0954579425101120.

## Pre-registration statement

This research was not pre-registered. This is a longitudinal study that began in 1986 and pre-registration was not operating at that time.
