# Supplementary material for: A prospective investigation of whether parent psychopathology explains the relationship between parent maltreatment and offspring mental health
Source: Dev Psychopathol. 2026 Jan 23:1–22. Online ahead of print. doi: 10.1017/S0954579425101120 (PMC12888080; doi:10.1017/S0954579425101120)
Supplement: Young et al. supplementary material [file S0954579425101120sup001.docx]

| **Supplementary Table 1. Correlations among study variables** | | | | | | | | | | | | | | | | |
| --- | --- | --- | --- | --- | --- | --- | --- | --- | --- | --- | --- | --- | --- | --- | --- | --- |
|  | *1* | *2* | *3* | *4* | *5* | *6* | *7* | *8* | *9* | *10* | *11* | *12* | *13* | *14* | *15* | *16* |
| 1. Parent childhood maltreatment | - |  |  |  |  |  |  |  |  |  |  |  |  |  |  |  |
| 2. Parent teenage pregnancy | 0.05 | - |  |  |  |  |  |  |  |  |  |  |  |  |  |  |
| 3. Parent depression | 0.08* | 0.08* | - |  |  |  |  |  |  |  |  |  |  |  |  |  |
| 4. Parent dysthymia | 0.13** | 0.09* | 0.88*** | - |  |  |  |  |  |  |  |  |  |  |  |  |
| 5. Parent anxiety | 0.05 | 0.07 | 0.55*** | 0.53*** | - |  |  |  |  |  |  |  |  |  |  |  |
| 6. Parent PTSD | 0.12** | 0.11** | 0.42*** | 0.43*** | 0.32*** | - |  |  |  |  |  |  |  |  |  |  |
| 7. Parent alcohol | 0.06 | 0.02 | 0.24*** | 0.22*** | 0.18*** | 0.21*** | - |  |  |  |  |  |  |  |  |  |
| 8. Parent drug | 0.04 | -0.00 | 0.28*** | 0.27*** | 0.24*** | 0.23*** | 0.49*** | - |  |  |  |  |  |  |  |  |
| 9. Minor offspring depression | 0.19* | 0.16* | 0.25** | 0.21** | -0.00 | 0.15 | 0.10 | 0.21** | - |  |  |  |  |  |  |  |
| 10. Minor offspring anxiety | 0.13 | 0.11 | 0.32*** | 0.24** | 0.12 | 0.14 | 0.11 | 0.15 | 0.64*** | - |  |  |  |  |  |  |
| 11. Minor offspring PTSD | 0.03 | 0.13 | 0.28** | 0.20* | 0.07 | 0.12 | 0.13 | 0.19* | 0.52*** | 0.62*** | - |  |  |  |  |  |
| 12. Adult offspring depression | 0.01 | 0.02 | 0.09 | 0.08 | 0.03 | -0.00 | -0.01 | 0.05 |  |  |  | - |  |  |  |  |
| 13. Adult offspring anxiety | -0.03 | -0.06 | 0.10* | 0.08 | 0.07 | 0.04 | 0.02 | 0.05 |  |  |  | 0.65*** | - |  |  |  |
| 14. Adult offspring PTSD | 0.07 | 0.04 | 0.11* | 0.15** | 0.06 | 0.03 | 0.11* | 0.07 |  |  |  | 0.38*** | 0.29*** | - |  |  |
| 15. Adult offspring alcohol | 0.07 | 0.04 | 0.16*** | 0.14*** | 0.12** | -0.01 | 0.17*** | 0.10* |  |  |  | 0.12** | 0.12* | 0.19*** | - |  |
| 16. Adult offspring drug | -0.03 | 0.10** | 0.16*** | 0.16*** | 0.14*** | 0.02 | 0.07 | 0.12** |  |  |  | 0.12** | 0.09* | 0.16** | 0.31*** | - |
| 17. Adult offspring marijuana | 0.03 | 0.08* | 0.17*** | 0.16*** | 0.17*** | 0.01 | 0.08* | 0.11** |  |  |  | 0.14** | 0.15** | 0.17*** | 0.34*** | 0.44*** |
| Spearman’s rank correlations and Pearson coefficients were used as appropriate.  ^*^*p* < 0.05, ^**^*p* < 0.01, ^***^*p* < 0.001. | | | | | | | | | | | | | | | | |

| **Supplementary Table 2.**  *Comparisons of parents with children who participated with parents with children who did not participate* | | | | | | | |
| --- | --- | --- | --- | --- | --- | --- | --- |
| **Variable** | **Parent**  **participated**  **N = 443** | | **Parent did not participate**  **N = 118** | |  |  |  |
|  | **N** | **%** | **N** | **%** | **Chi square** | ***p*** | **Odds**  **Ratio** |
| Maltreated | 238 | 53.7 | 66 | 55.9 | 0.10 | .746 | 0.91 |
| Female | 279 | 63.0 | 41 | 34.7 | 29.17 | **<.001** | 3.19 |
| White | 257 | 58.0 | 72 | 61.0 | 0.23 | .629 | 0.88 |
| Teenage parent | 156 | 35.2 | 33 | 28.0 | 1.88 | .140 | 1.40 |
|  | **Mean** | **SD** | **Mean** | **SD** | **T score** | ***p*** | **Cohen’s d** |
| Age at interview 1 | 28.91 | 3.74 | 28.56 | 3.87 | 0.91 | .363 | 0.09 |
|  |  |  |  |  |  |  |  |
| Psychiatric Symptoms | **Mean** | **SD** | **Mean** | **SD** | **T score** | ***p*** |  |
| Major depressive disorder | 3.58 | 2.72 | 3.50 | 2.86 | 0.27 | .791 | 0.03 |
| Dysthymia | 2.55 | 2.09 | 2.53 | 2.21 | 0.13 | .900 | 0.01 |
| Posttraumatic stress disorder | 5.57 | 5.88 | 5.65 | 5.81 | 0.13 | .898 | 0.01 |
| Generalized anxiety disorder | 4.28 | 5.12 | 4.38 | 5.29 | 0.19 | .849 | 0.02 |
| Alcohol abuse/dependence | 2.25 | 2.60 | 3.35 | 2.76 | 4.04 | **<.001** | 0.42 |
| Drug abuse/dependence | 1.69 | 2.51 | 1.92 | 2.51 | 0.88 | .377 | 0.09 |
| Notes: SD = standard deviation. Psychiatric symptoms represent the number of lifetime symptoms based on the NIMH Diagnostic Interview Schedule III-R. | | | | | | | |

| **Supplementary Table 3**  *Results of structural equation models testing whether parent internalizing psychopathology and substance use mediates the relationship between childhood maltreatment and minor offspring symptoms* | | | | | | | | | |
| --- | --- | --- | --- | --- | --- | --- | --- | --- | --- |
|  | **Est.** | **SE** | **95% CI** | ***p*** |  | **χ ^2^** | **CFI** | **TLI** | **RMSEA** |
| ***Total sample*** |  |  |  |  |  |  |  |  |  |
| Total effect | 0.76 | 0.41 | 0.09, 1.43 | .062 |  | 103.267*** | 0.901 | 0.839 | 0.093 |
|  |  |  |  |  |  |  |  |  |  |
| Parent CM -> Minor offspring depression | 0.41 | 0.15 | 0.18, 0.65 | **.004** |  |  |  |  |  |
| Parent CM -> Minor offspring anxiety | 0.22 | 0.16 | -0.05, 0.49 | .175 |  |  |  |  |  |
| Parent CM -> Minor offspring PTSD | 0.07 | 0.17 | -0.21, 0.36 | .671 |  |  |  |  |  |
|  |  |  |  |  |  |  |  |  |  |
| Parent internalizing -> Minor offspring depression | 0.11 | 0.11 | -0.06, 0.29 | .283 |  |  |  |  |  |
| Parent internalizing -> Minor offspring anxiety | 0.22 | 0.10 | 0.05, 0.38 | **.034** |  |  |  |  |  |
| Parent internalizing -> Minor offspring PTSD | 0.11 | 0.11 | -0.07, 0.29 | .301 |  |  |  |  |  |
| Parent substance -> Minor offspring depression | 0.40 | 0.22 | 0.05, 0.75 | .062 |  |  |  |  |  |
| Parent substance -> Minor offspring anxiety | 0.27 | 0.16 | -0.00, 0.54 | .101 |  |  |  |  |  |
| Parent substance -> Minor offspring PTSD | 0.17 | 0.18 | -0.13, 0.46 | .352 |  |  |  |  |  |
|  |  |  |  |  |  |  |  |  |  |
| Parent CM -> Parent internalizing | 0.30 | 0.16 | 0.03, 0.56 | .067 |  |  |  |  |  |
| Parent CM -> Parent substance | -0.10 | 0.17 | -0.38, 0.19 | .572 |  |  |  |  |  |
|  |  |  |  |  |  |  |  |  |  |
| Parent CM -> Parent internalizing -> Minor offspring depression | 0.03 | 0.04 | -0.03, 0.09 | .361 |  |  |  |  |  |
| Parent CM -> Parent internalizing -> Minor offspring anxiety | 0.06 | 0.05 | -0.01, 0.14 | .180 |  |  |  |  |  |
| Parent CM -> Parent internalizing -> Minor offspring PTSD | 0.03 | 0.04 | -0.03, 0.09 | .377 |  |  |  |  |  |
| Parent CM -> Parent substance use -> Minor offspring depression | -0.04 | 0.06 | -0.15, 0.07 | .541 |  |  |  |  |  |
| Parent CM -> Parent substance use -> Minor offspring anxiety | -0.03 | 0.05 | -0.10, 0.05 | .565 |  |  |  |  |  |
| Parent CM -> Parent substance use -> Minor offspring PTSD | -0.02 | 0.03 | -0.06, 0.03 | .561 |  |  |  |  |  |
|  |  |  |  |  |  |  |  |  |  |
| Total indirect effect | 0.05 | 0.20 | -0.28, 0.37 | .810 |  |  |  |  |  |
|  |  |  |  |  |  |  |  |  |  |
| ***Female Parents*** |  |  |  |  |  |  |  |  |  |
| Total effect | 0.52 | 0.59 | -0.45, 1.49 | .375 |  | 76.842** | 0.904 | 0.844 | 0.093 |
|  |  |  |  |  |  |  |  |  |  |
| Parent CM -> Minor offspring depression | 0.45 | 0.18 | 0.16, 0.75 | **.012** |  |  |  |  |  |
| Parent CM -> Minor offspring anxiety | -0.00 | 0.24 | -0.40, 0.39 | .987 |  |  |  |  |  |
| Parent CM -> Minor offspring PTSD | -0.07 | 0.22 | -0.43, 0.29 | .740 |  |  |  |  |  |
|  |  |  |  |  |  |  |  |  |  |
| Parent internalizing -> Minor offspring depression | 0.02 | 0.14 | -0.21, 0.24 | .897 |  |  |  |  |  |
| Parent internalizing -> Minor offspring anxiety | 0.25 | 0.14 | 0.02, 0.48 | .076 |  |  |  |  |  |
| Parent internalizing -> Minor offspring PTSD | 0.12 | 0.15 | -0.13, 0.36 | .437 |  |  |  |  |  |
| Parent substance -> Minor offspring depression | 0.62 | 0.23 | 0.24, 0.99 | **.007** |  |  |  |  |  |
| Parent substance -> Minor offspring anxiety | 0.50 | 0.17 | 0.22, 0.77 | **.003** |  |  |  |  |  |
| Parent substance -> Minor offspring PTSD | 0.41 | 0.24 | 0.01, 0.81 | .095 |  |  |  |  |  |
|  |  |  |  |  |  |  |  |  |  |
| Parent CM -> Parent internalizing | 0.38 | 0.24 | -0.02, 0.77 | .120 |  |  |  |  |  |
| Parent CM -> Parent substance | 0.00 | 0.24 | -0.40, 0.40 | .997 |  |  |  |  |  |
|  |  |  |  |  |  |  |  |  |  |
| Parent CM -> Parent internalizing -> Minor offspring depression | 0.01 | 0.05 | -0.08, 0.09 | .898 |  |  |  |  |  |
| Parent CM -> Parent internalizing -> Minor offspring anxiety | 0.09 | 0.08 | -0.04, 0.22 | .246 |  |  |  |  |  |
| Parent CM -> Parent internalizing -> Minor offspring PTSD | 0.04 | 0.06 | -0.06, 0.15 | .496 |  |  |  |  |  |
| Parent CM -> Parent substance use -> Minor offspring depression | 0.00 | 0.15 | -0.24, 0.25 | .997 |  |  |  |  |  |
| Parent CM -> Parent substance use -> Minor offspring anxiety | 0.00 | 0.12 | -0.20, 0.20 | .997 |  |  |  |  |  |
| Parent CM -> Parent substance use -> Minor offspring PTSD | 0.00 | 0.10 | -0.16, 0.16 | .997 |  |  |  |  |  |
|  |  |  |  |  |  |  |  |  |  |
| Total indirect effect | 0.14 | 0.45 | -0.59, 0.88 | .746 |  |  |  |  |  |
|  |  |  |  |  |  |  |  |  |  |
| ***Male Parents*** |  |  |  |  |  |  |  |  |  |
| Total effect | 1.05 | 0.53 | 0.18, 1.92 | **.046** |  | 75.549** | 0.886 | 0.813 | 0.100 |
|  |  |  |  |  |  |  |  |  |  |
| Parent CM -> Minor offspring depression | 0.31 | 0.20 | -0.02, 0.65 | .126 |  |  |  |  |  |
| Parent CM -> Minor offspring anxiety | 0.43 | 0.19 | 0.12, 0.74 | **.022** |  |  |  |  |  |
| Parent CM -> Minor offspring PTSD | 0.18 | 0.27 | -0.26, 0.62 | .504 |  |  |  |  |  |
|  |  |  |  |  |  |  |  |  |  |
| Parent internalizing -> Minor offspring depression | 0.28 | 0.09 | 0.13, 0.42 | **.002** |  |  |  |  |  |
| Parent internalizing -> Minor offspring anxiety | 0.15 | 0.11 | -0.03, 0.33 | .181 |  |  |  |  |  |
| Parent internalizing -> Minor offspring PTSD | 0.03 | 0.13 | -0.19, 0.25 | .816 |  |  |  |  |  |
| Parent substance -> Minor offspring depression | -0.01 | 0.05 | -0.08, 0.07 | .903 |  |  |  |  |  |
| Parent substance -> Minor offspring anxiety | -0.03 | 0.08 | -0.15, 0.10 | .745 |  |  |  |  |  |
| Parent substance -> Minor offspring PTSD | -0.07 | 0.18 | -0.37, 0.22 | .689 |  |  |  |  |  |
|  |  |  |  |  |  |  |  |  |  |
| Parent CM -> Parent internalizing | 0.21 | 0.23 | -0.16, 0.59 | .354 |  |  |  |  |  |
| Parent CM -> Parent substance | -0.30 | 0.21 | -0.65, 0.05 | .152 |  |  |  |  |  |
|  |  |  |  |  |  |  |  |  |  |
| Parent CM -> Parent internalizing -> Minor offspring depression | 0.06 | 0.06 | -0.05, 0.16 | .366 |  |  |  |  |  |
| Parent CM -> Parent internalizing -> Minor offspring anxiety | 0.03 | 0.04 | -0.04, 0.10 | .437 |  |  |  |  |  |
| Parent CM -> Parent internalizing -> Minor offspring PTSD | 0.01 | 0.03 | -0.04, 0.05 | .820 |  |  |  |  |  |
| Parent CM -> Parent substance use -> Minor offspring depression | 0.00 | 0.02 | -0.02, 0.03 | .904 |  |  |  |  |  |
| Parent CM -> Parent substance use -> Minor offspring anxiety | 0.01 | 0.02 | -0.03, 0.04 | .730 |  |  |  |  |  |
| Parent CM -> Parent substance use -> Minor offspring PTSD | 0.02 | 0.05 | -0.06, 0.10 | .663 |  |  |  |  |  |
|  |  |  |  |  |  |  |  |  |  |
| Total indirect effect | 0.13 | 0.13 | -0.08, 0.33 | .313 |  |  |  |  |  |
|  |  |  |  |  |  |  |  |  |  |
| Notes: Results from SEM models testing for mediation with two-factor parent models. SE = standard error; CI = confidence interval; χ ^2^ = critical ratio chi square; CFI = comparative fit index; TLI =Tucker-Lewis index; RMSEA = root mean square error of approximation; CM = childhood maltreatment. Offspring and parent psychiatric symptom variables are all standardized. Offspring anxiety and depression represent current symptoms. Offspring PTSD represent lifetime symptoms. All analyses controlled for offsprings’ sex and race on the offspring symptoms. Teenage pregnancy (first pregnancy was when the parent was less than 20 years old) is controlled for on the paths from parent maltreatment to parent symptoms. ***p*<.01. ****p*<.001 | | | | | | | | | |

| **Supplementary Table 4**  *Results of structural equation models testing whether parent internalizing psychopathology and substance use mediates the relationship between childhood maltreatment and adult offspring symptoms* | | | | | | | | | |
| --- | --- | --- | --- | --- | --- | --- | --- | --- | --- |
|  | **Est.** | **SE** | **95% CI** | ***p*** |  | **χ ^2^** | **CFI** | **TLI** | **RMSEA** |
| ***Total sample*** |  |  |  |  |  |  |  |  |  |
| Total effect | 0.16 | 0.36 | -0.43, 0.76 | .651 |  | 114.871*** | 0.967 | 0.936 | 0.042 |
|  |  |  |  |  |  |  |  |  |  |
| Parent CM -> Adult offspring depression | -0.01 | 0.09 | -0.16, 0.14 | .944 |  |  |  |  |  |
| Parent CM -> Adult offspring anxiety | -0.09 | 0.09 | -0.24, 0.06 | .317 |  |  |  |  |  |
| Parent CM -> Adult offspring PTSD | 0.08 | 0.09 | -0.07, 0.24 | .379 |  |  |  |  |  |
| Parent CM -> Adult offspring alcohol | 0.08 | 0.10 | -0.08, 0.24 | .386 |  |  |  |  |  |
| Parent CM -> Adult offspring drug | -0.12 | 0.10 | -0.28, 0.05 | .245 |  |  |  |  |  |
| Parent CM -> Adult offspring marijuana | 0.04 | 0.09 | -0.11, 0.19 | .661 |  |  |  |  |  |
|  |  |  |  |  |  |  |  |  |  |
| Parent internalizing -> Adult offspring depression | 0.11 | 0.05 | 0.02, 0.19 | **.035** |  |  |  |  |  |
| Parent internalizing -> Adult offspring anxiety | 0.10 | 0.05 | 0.01, 0.18 | .077 |  |  |  |  |  |
| Parent internalizing -> Adult offspring PTSD | 0.11 | 0.06 | 0.02, 0.20 | **.048** |  |  |  |  |  |
| Parent internalizing -> Adult offspring alcohol | 0.14 | 0.06 | 0.05, 0.24 | **.016** |  |  |  |  |  |
| Parent internalizing -> Adult offspring drug | 0.20 | 0.07 | 0.08, 0.31 | **.005** |  |  |  |  |  |
| Parent internalizing -> Adult offspring marijuana | 0.21 | 0.06 | 0.11, 0.31 | **.001** |  |  |  |  |  |
| Parent substance -> Adult offspring depression | -0.05 | 0.04 | -0.12, 0.02 | .235 |  |  |  |  |  |
| Parent substance -> Adult offspring anxiety | -0.01 | 0.04 | -0.08, 0.05 | .753 |  |  |  |  |  |
| Parent substance -> Adult offspring PTSD | 0.05 | 0.05 | -0.04, 0.13 | .371 |  |  |  |  |  |
| Parent substance -> Adult offspring alcohol | 0.13 | 0.11 | -0.05, 0.31 | .225 |  |  |  |  |  |
| Parent substance -> Adult offspring drug | 0.03 | 0.06 | -0.07, 0.13 | .644 |  |  |  |  |  |
| Parent substance -> Adult offspring marijuana | 0.01 | 0.06 | -0.10, 0.11 | .924 |  |  |  |  |  |
|  |  |  |  |  |  |  |  |  |  |
| Parent CM -> Parent internalizing | 0.17 | 0.11 | -0.01, 0.35 | .117 |  |  |  |  |  |
| Parent CM -> Parent substance | 0.18 | 0.11 | -0.01, 0.36 | .123 |  |  |  |  |  |
|  |  |  |  |  |  |  |  |  |  |
| Parent CM -> Parent internalizing -> Adult offspring depression | 0.02 | 0.02 | -0.01, 0.04 | .210 |  |  |  |  |  |
| Parent CM -> Parent internalizing -> Adult offspring anxiety | 0.02 | 0.01 | -0.01, 0.04 | .244 |  |  |  |  |  |
| Parent CM -> Parent internalizing -> Adult offspring PTSD | 0.02 | 0.02 | -0.01, 0.04 | .222 |  |  |  |  |  |
| Parent CM -> Parent internalizing -> Adult offspring alcohol | 0.02 | 0.02 | -0.01, 0.05 | .173 |  |  |  |  |  |
| Parent CM -> Parent internalizing -> Adult offspring drug | 0.03 | 0.02 | -0.01, 0.07 | .172 |  |  |  |  |  |
| Parent CM -> Parent internalizing -> Adult offspring marijuana | 0.04 | 0.03 | -0.01, 0.08 | .151 |  |  |  |  |  |
| Parent CM -> Parent substance use -> Adult offspring depression | -0.01 | 0.01 | -0.02, 0.01 | .372 |  |  |  |  |  |
| Parent CM -> Parent substance use -> Adult offspring anxiety | -0.00 | 0.01 | -0.01, 0.01 | .763 |  |  |  |  |  |
| Parent CM -> Parent substance use -> adult offspring PTSD | 0.01 | 0.01 | -0.01, 0.03 | .452 |  |  |  |  |  |
| Parent CM -> Parent substance use -> adult offspring alcohol | 0.02 | 0.03 | -0.02, 0.07 | .374 |  |  |  |  |  |
| Parent CM -> Parent substance use -> adult offspring drug | 0.01 | 0.01 | -0.01, 0.02 | .662 |  |  |  |  |  |
| Parent CM -> Parent substance use -> adult offspring marijuana | 0.00 | 0.01 | -0.02, 0.02 | .925 |  |  |  |  |  |
|  |  |  |  |  |  |  |  |  |  |
| Total indirect effect | 0.17 | 0.11 | -0.01, 0.35 | .120 |  |  |  |  |  |
|  |  |  |  |  |  |  |  |  |  |
| ***Female Parents*** |  |  |  |  |  |  |  |  |  |
| Total effect | 0.26 | 0.47 | -0.51, 1.02 | .579 |  | 133.861*** | 0.936 | 0.877 | 0.060 |
|  |  |  |  |  |  |  |  |  |  |
| Parent CM -> Adult offspring depression | -0.13 | 0.12 | -0.33, 0.06 | .252 |  |  |  |  |  |
| Parent CM -> Adult offspring anxiety | -0.12 | 0.12 | -0.31, 0.07 | .313 |  |  |  |  |  |
| Parent CM -> Adult offspring PTSD | 0.17 | 0.12 | -0.03, 0.37 | .160 |  |  |  |  |  |
| Parent CM -> Adult offspring alcohol | 0.13 | 0.12 | -0.06, 0.32 | .273 |  |  |  |  |  |
| Parent CM -> Adult offspring drug | -0.08 | 0.13 | -0.30, 0.14 | .531 |  |  |  |  |  |
| Parent CM -> Adult offspring marijuana | 0.14 | 0.12 | -0.05, 0.33 | .234 |  |  |  |  |  |
|  |  |  |  |  |  |  |  |  |  |
| Parent internalizing -> Adult offspring depression | 0.08 | 0.07 | -0.04, 0.19 | .278 |  |  |  |  |  |
| Parent internalizing -> Adult offspring anxiety | 0.07 | 0.07 | -0.04, 0.18 | .289 |  |  |  |  |  |
| Parent internalizing -> Adult offspring PTSD | 0.08 | 0.07 | -0.04, 0.19 | .260 |  |  |  |  |  |
| Parent internalizing -> Adult offspring alcohol | 0.19 | 0.08 | 0.06, 0.32 | **.016** |  |  |  |  |  |
| Parent internalizing -> Adult offspring drug | 0.18 | 0.10 | 0.02, 0.34 | .073 |  |  |  |  |  |
| Parent internalizing -> Adult offspring marijuana | 0.29 | 0.08 | 0.15, 0.42 | **.001** |  |  |  |  |  |
| Parent substance -> Adult offspring depression | -0.02 | 0.03 | -0.07, 0.03 | .601 |  |  |  |  |  |
| Parent substance -> Adult offspring anxiety | 0.02 | 0.04 | -0.05, 0.09 | .702 |  |  |  |  |  |
| Parent substance -> Adult offspring PTSD | 0.05 | 0.07 | -0.07, 0.17 | .517 |  |  |  |  |  |
| Parent substance -> Adult offspring alcohol | 0.05 | 0.10 | -0.12, 0.22 | .634 |  |  |  |  |  |
| Parent substance -> Adult offspring drug | 0.02 | 0.07 | -0.09, 0.14 | .760 |  |  |  |  |  |
| Parent substance -> Adult offspring marijuana | -0.03 | 0.03 | -0.08, 0.03 | .464 |  |  |  |  |  |
|  |  |  |  |  |  |  |  |  |  |
| Parent CM -> Parent internalizing | 0.15 | 0.14 | -0.08, 0.38 | .271 |  |  |  |  |  |
| Parent CM -> Parent substance | 0.24 | 0.15 | 0.00, 0.48 | .096 |  |  |  |  |  |
|  |  |  |  |  |  |  |  |  |  |
| Parent CM -> Parent internalizing -> Adult offspring depression | 0.01 | 0.02 | -0.01, 0.04 | .454 |  |  |  |  |  |
| Parent CM -> Parent internalizing -> Adult offspring anxiety | 0.01 | 0.01 | -0.01, 0.03 | .448 |  |  |  |  |  |
| Parent CM -> Parent internalizing -> Adult offspring PTSD | 0.01 | 0.02 | -0.01, 0.04 | .445 |  |  |  |  |  |
| Parent CM -> Parent internalizing -> Adult offspring alcohol | 0.03 | 0.03 | -0.02, 0.07 | .302 |  |  |  |  |  |
| Parent CM -> Parent internalizing -> Adult offspring drug | 0.03 | 0.03 | -0.02, 0.08 | .361 |  |  |  |  |  |
| Parent CM -> Parent internalizing -> Adult offspring marijuana | 0.04 | 0.04 | -0.02, 0.11 | .291 |  |  |  |  |  |
| Parent CM -> Parent substance use -> Adult offspring depression | -0.00 | 0.01 | -0.02, 0.01 | .638 |  |  |  |  |  |
| Parent CM -> Parent substance use -> Adult offspring anxiety | 0.00 | 0.01 | -0.02, 0.02 | .734 |  |  |  |  |  |
| Parent CM -> Parent substance use -> adult offspring PTSD | 0.01 | 0.02 | -0.03, 0.05 | .613 |  |  |  |  |  |
| Parent CM -> Parent substance use -> adult offspring alcohol | 0.01 | 0.03 | -0.04, 0.06 | .688 |  |  |  |  |  |
| Parent CM -> Parent substance use -> adult offspring drug | 0.01 | 0.02 | -0.03, 0.04 | .779 |  |  |  |  |  |
| Parent CM -> Parent substance use -> adult offspring marijuana | -0.01 | 0.01 | -0.02, 0.01 | .512 |  |  |  |  |  |
|  |  |  |  |  |  |  |  |  |  |
| Total indirect effect | 0.16 | 0.15 | -0.09, 0.40 | .305 |  |  |  |  |  |
|  |  |  |  |  |  |  |  |  |  |
| ***Male Parents*** |  |  |  |  |  |  |  |  |  |
| Total effect | -0.18 | 0.54 | -1.07, 0.71 | .741 |  | 80.840* | 0.963 | 0.929 | 0.045 |
|  |  |  |  |  |  |  |  |  |  |
| Parent CM -> Adult offspring depression | 0.22 | 0.14 | -0.02, 0.44 | .124 |  |  |  |  |  |
| Parent CM -> Adult offspring anxiety | -0.08 | 0.14 | -0.31, 0.15 | .575 |  |  |  |  |  |
| Parent CM -> Adult offspring PTSD | -0.10 | 0.16 | -0.36, 0.16 | .523 |  |  |  |  |  |
| Parent CM -> Adult offspring alcohol | -0.01 | 0.15 | -0.25, 0.24 | .973 |  |  |  |  |  |
| Parent CM -> Adult offspring drug | -0.17 | 0.13 | -0.39, 0.05 | .205 |  |  |  |  |  |
| Parent CM -> Adult offspring marijuana | -0.15 | 0.14 | -0.15, 0.08 | .278 |  |  |  |  |  |
|  |  |  |  |  |  |  |  |  |  |
| Parent internalizing -> Adult offspring depression | 0.16 | 0.09 | 0.02, 0.30 | .063 |  |  |  |  |  |
| Parent internalizing -> Adult offspring anxiety | 0.08 | 0.11 | -0.09, 0.26 | .432 |  |  |  |  |  |
| Parent internalizing -> Adult offspring PTSD | 0.12 | 0.10 | -0.05, 0.28 | .258 |  |  |  |  |  |
| Parent internalizing -> Adult offspring alcohol | 0.03 | 0.08 | -0.10, 0.16 | .704 |  |  |  |  |  |
| Parent internalizing -> Adult offspring drug | 0.19 | 0.10 | 0.03, 0.36 | .052 |  |  |  |  |  |
| Parent internalizing -> Adult offspring marijuana | 0.04 | 0.07 | -0.08, 0.16 | .580 |  |  |  |  |  |
| Parent substance -> Adult offspring depression | -0.08 | 0.08 | -0.22, 0.06 | .359 |  |  |  |  |  |
| Parent substance -> Adult offspring anxiety | -0.03 | 0.08 | -0.17, 0.10 | .697 |  |  |  |  |  |
| Parent substance -> Adult offspring PTSD | 0.08 | 0.11 | -0.10, 0.26 | .479 |  |  |  |  |  |
| Parent substance -> Adult offspring alcohol | 0.27 | 0.12 | 0.07, 0.46 | **.024** |  |  |  |  |  |
| Parent substance -> Adult offspring drug | 0.06 | 0.11 | -0.13, 0.25 | .601 |  |  |  |  |  |
| Parent substance -> Adult offspring marijuana | 0.07 | 0.08 | -0.06, 0.20 | .387 |  |  |  |  |  |
|  |  |  |  |  |  |  |  |  |  |
| Parent CM -> Parent internalizing | 0.12 | 0.18 | -0.18, 0.42 | .505 |  |  |  |  |  |
| Parent CM -> Parent substance | 0.10 | 0.20 | -0.24, 0.43 | .632 |  |  |  |  |  |
|  |  |  |  |  |  |  |  |  |  |
| Parent CM -> Parent internalizing -> Adult offspring depression | 0.02 | 0.03 | -0.03, 0.07 | .513 |  |  |  |  |  |
| Parent CM -> Parent internalizing -> Adult offspring anxiety | 0.23 | 0.15 | -0.02, 0.04 | .117 |  |  |  |  |  |
| Parent CM -> Parent internalizing -> Adult offspring PTSD | 0.01 | 0.02 | -0.02, 0.05 | .538 |  |  |  |  |  |
| Parent CM -> Parent internalizing -> Adult offspring alcohol | 0.00 | 0.01 | -0.01, 0.02 | .739 |  |  |  |  |  |
| Parent CM -> Parent internalizing -> Adult offspring drug | 0.02 | 0.04 | -0.04, 0.09 | .528 |  |  |  |  |  |
| Parent CM -> Parent internalizing -> Adult offspring marijuana | 0.01 | 0.01 | -0.02, 0.03 | .685 |  |  |  |  |  |
| Parent CM -> Parent substance use -> Adult offspring depression | -0.01 | 0.02 | -0.04, 0.02 | .674 |  |  |  |  |  |
| Parent CM -> Parent substance use -> Adult offspring anxiety | -0.00 | 0.01 | -0.02, 0.01 | .766 |  |  |  |  |  |
| Parent CM -> Parent substance use -> adult offspring PTSD | 0.01 | 0.02 | -0.02, 0.04 | .668 |  |  |  |  |  |
| Parent CM -> Parent substance use -> adult offspring alcohol | 0.03 | 0.05 | -0.06, 0.11 | .628 |  |  |  |  |  |
| Parent CM -> Parent substance use -> adult offspring drug | 0.01 | 0.02 | -0.02, 0.03 | .691 |  |  |  |  |  |
| Parent CM -> Parent substance use -> adult offspring marijuana | 0.01 | 0.02 | -0.02, 0.03 | .667 |  |  |  |  |  |
|  |  |  |  |  |  |  |  |  |  |
| Total indirect effect | 0.11 | 0.15 | -0.14, 0.36 | .467 |  |  |  |  |  |
|  |  |  |  |  |  |  |  |  |  |
| Notes: Results from SEM models testing for mediation with two-factor parent models. SE = standard error; CI = confidence interval; χ ^2^ = critical ratio chi square; CFI = comparative fit index; TLI =Tucker-Lewis index; RMSEA = root mean square error of approximation; CM = childhood maltreatment. Offspring and parent psychiatric symptom variables are all standardized. Offspring anxiety and depression represent current symptoms. Offspring PTSD, alcohol use, drug use, and marijuana use represent lifetime symptoms. All analyses controlled for offsprings’ sex and race on the offspring symptoms. Teenage pregnancy (first pregnancy was when the parent was less than 20 years old) is controlled for on the paths from parent maltreatment to parent symptoms. **p*<.05. ****p*<.001 | | | | | | | | | |

| **Supplementary Table 5**  *Results of regressions testing whether parent sex and offspring age moderate the relationship between parent maltreatment history and offspring psychiatric symptoms* | | | | | | | | | | | | | | | | | | | | | | | | | | | | | | | | |
| --- | --- | --- | --- | --- | --- | --- | --- | --- | --- | --- | --- | --- | --- | --- | --- | --- | --- | --- | --- | --- | --- | --- | --- | --- | --- | --- | --- | --- | --- | --- | --- | --- |
|  | **Offspring** | | | | | | | | | | | | | | | | | | | | | | | | | | | | | | | |
|  | **Depression** | | | | | **Anxiety** | | | | | **PTSD** | | | | | **Alcohol (Adult only)** | | | | | **Drug (Adult only)** | | | | | | **Marijuana (Adult only)** | | | | | |
|  | **Est.** | **SE** | **β** | ***p*** | **adj.  *p*** | **Est.** | **SE** | **β** | ***p*** | **adj.  *p*** | **Est.** | **SE** | **β** | ***p*** | **adj.  *p*** | **Est.** | **SE** | **β** | ***p*** | **adj.**  ***p*** | **Est.** | **SE** | **β** | ***p*** | **adj.  *p*** | **Est.** | | **SE** | **β** | ***p*** | **adj.  *p*** |  |
| Group: Maltreated | 0.15 | 0.33 | 0.08 | .644 | .952 | -0.04 | 0.33 | -0.02 | .911 | .952 | 0.29 | 0.31 | 0.15 | .347 | .952 | -0.08 | 0.29 | -0.04 | .777 | .952 | -0.15 | 0.36 | -0.07 | .683 | .952 | 0.04 | | 0.35 | 0.02 | .915 | .952 |  |
| Sex: Female | 0.30 | 0.32 | 0.15 | .341 | .952 | 0.08 | 0.31 | 0.04 | .795 | .952 | 0.38 | 0.303 | 0.18 | .208 | .952 | 0.14 | 0.30 | 0.07 | .642 | .952 | -0.75 | 0.31 | -0.36 | .016 | .288 | -0.37 | | 0.31 | -0.18 | .237 | .952 |  |
| Group x Sex | -0.01 | 0.20 | -0.01 | .952 | .952 | 0.16 | 0.20 | 0.18 | .425 | .952 | -0.13 | 0.19 | -0.15 | .504 | .952 | 0.17 | 0.19 | 0.20 | .360 | .952 | 0.17 | 0.20 | 0.20 | .398 | .952 | 0.03 | | 0.20 | 0.04 | .868 | .952 |  |
| R^2^ | 0.037 |  |  |  |  | 0.052 |  |  |  |  | 0.017 |  |  |  |  | 0.056 |  |  |  |  | 0.082 |  |  |  |  | 0.041 | |  |  |  |  |  |
|  |  |  |  |  |  |  |  |  |  |  |  |  |  |  |  |  |  |  |  |  |  |  |  |  |  |  | |  |  |  |  |  |
| Group: Maltreated | 0.78 | 0.30 | 0.39 | .009 | .081 | 0.65 | 0.31 | 0.33 | .032 | .151 | 0.15 | 0.30 | 0.07 | .629 | .782 | 0.28 | 0.31 | 0.14 | .364 | .645 | -0.34 | 0.28 | -0.17 | .224 | .448 | -0.06 | | 0.31 | -0.03 | .850 | .861 |  |
| Offspring Age | 0.06 | 0.02 | 0.36 | .007 | .081 | 0.04 | 0.02 | 0.28 | .042 | .151 | 0.01 | 0.02 | 0.08 | .544 | .753 | 0.01 | 0.02 | 0.09 | .508 | .753 | -0.03 | 0.02 | -0.16 | .191 | .430 | -0.02 | | 0.02 | -0.11 | .394 | .645 |  |
| Group x Age | -0.03 | 0.01 | -0.43 | .035 | .151 | -0.02 | 0.01 | -0.29 | .164 | .422 | -0.00 | 0.01 | -0.04 | .861 | .861 | -0.00 | 0.01 | -0.04 | .832 | .861 | 0.02 | 0.01 | 0.31 | .110 | .330 | 0.01 | | 0.01 | 0.09 | .652 | .782 |  |
| R^2^ | 0.026 |  |  |  |  | 0.031 |  |  |  |  | 0.008 |  |  |  |  | 0.019 |  |  |  |  | 0.029 |  |  |  |  | 0.018 | |  |  |  |  |  |
| Note: Est. = Estimate; SE = standard error; β = standardized regression coefficient; Adj. p = p-value adjusted using the false discovery rate (FDR) correction; DEP = depression; GAD = generalized anxiety disorder; PTSD = posttraumatic stress disorder; ALC = alcohol abuse and/or dependence; DRG = drug abuse and/or dependence. Sex represents the sex of the parent. Offspring variables were standardized. Robust standard errors are reported. Analyses control for offsprings’ sex and race; the first model testing parent sex as a moderator additionally controls for offsprings’ age. | | | | | | | | | | | | | | | | | | | | | | | | | | | | | | | | |
